# Efficacy of Short-Term High-Dose Statin Pretreatment in Prevention of Contrast-Induced Acute Kidney Injury: Updated Study-Level Meta-Analysis of 13 Randomized Controlled Trials

**DOI:** 10.1371/journal.pone.0111397

**Published:** 2014-11-04

**Authors:** Joo Myung Lee, Jonghanne Park, Ki-Hyun Jeon, Ji-hyun Jung, Sang Eun Lee, Jung-Kyu Han, Hack-Lyoung Kim, Han-Mo Yang, Kyung Woo Park, Hyun-Jae Kang, Bon-Kwon Koo, Sang-Ho Jo, Hyo-Soo Kim

**Affiliations:** 1 Department of Internal Medicine and Cardiovascular Center, Seoul National University Hospital, Seoul, Korea; 2 Cardiovascular Center, Seoul National University, Boramae Medical Center, Seoul, Korea; 3 Division of Cardiology, Department of Internal Medicine, Hallym University Sacred Heart Hospital, Anyang-si, Gyeonggi-do, Korea; 4 Department of Molecular Medicine and Biopharmaceutical Sciences, Graduate School of Convergence Science and Technology, Seoul National University, Seoul, Korea; Mario Negri Institute for Pharmacological Research and Azienda Ospedaliera Ospedali Riuniti di Bergamo, Italy

## Abstract

**Background:**

There have been conflicting results across the trials that evaluated prophylactic efficacy of short-term high-dose statin pre-treatment for prevention of contrast-induced acute kidney injury (CIAKI) in patients undergoing coronary angiography (CAG). The aim of the study was to perform an up-to-date meta-analysis regarding the efficacy of high-dose statin pre-treatment in preventing CIAKI.

**Methods and Results:**

Randomized-controlled trials comparing high-dose statin versus low-dose statin or placebo pre-treatment for prevention of CIAKI in patients undergoing CAG were included. The primary endpoint was the incidence of CIAKI within 2–5days after CAG. The relative risk (RR) with 95% CI was the effect measure. This analysis included 13 RCTs with 5,825 total patients; about half of them (n = 2,889) were pre-treated with high-dose statin (at least 40 mg of atorvastatin) before CAG, and the remainders (n = 2,936) pretreated with low-dose statin or placebo. In random-effects model, high-dose statin pre-treatment significantly reduced the incidence of CIAKI (RR 0.45, 95% CI 0.35–0.57, p<0.001, I^2^ = 8.2%, NNT 16), compared with low-dose statin or placebo. The benefit of high-dose statin was consistent in both comparisons with low-dose statin (RR 0.47, 95% CI 0.34–0.65, p<0.001, I^2^ = 28.4%, NNT 19) or placebo (RR 0.34, 95% CI 0.21–0.58, p<0.001, I^2^ = 0.0%, NNT 16). In addition, high-dose statin showed significant reduction of CIAKI across various subgroups of chronic kidney disease, acute coronary syndrome, and old age (≥60years), regardless of osmolality of contrast or administration of N-acetylcystein.

**Conclusions:**

High-dose statin pre-treatment significantly reduced overall incidence of CIAKI in patients undergoing CAG, and emerges as an effective prophylactic measure to prevent CIAKI.

## Introduction

Contrast-induced acute kidney injury (CIAKI) is a well-recognized complication of coronary angiography (CAG) with iodinated contrast medium and is the third leading cause of hospital-acquired acute renal failure. CIAKI has been known to be associated with prolonged hospitalization, increased costs, and increased short and long-term morbidity and mortality. [1] The incidence of CIAKI varies widely depending on the patient's underlying co-morbidities, definition criteria, and preventive strategies. But, certain subgroup of coronary heart disease patients, especially with acute coronary syndrome or chronic kidney disease, showed higher risk for the CIAKI. [2], [3] Investigators have examined several strategies to prevent CIAKI, such as fenolopam, mannitol, theophylline, iloprost, furosemide, dopamine, hemofiltration, ascorbic acid, and N-acetylcystein (NAC). [4] However, none of the agents were proved to be effective in preventing CIAKI. [4], [5] Currently, recommendations of the European Society of Cardiology/European Association for Cardio-Thoracic Surgery (ESC/EACTS) or the ACCF/AHA/SCAI guideline are limited to the prophylactic intravenous hydration, use of iso- or low-osmolar contrast agents, and reduced dosages of contrast agents to prevent occurrence of CIAKI. [6], [7] Since a few observational studies suggested that 3-hydroxyl-3-methylglutaryl coenzyme A reductase inhibitors (statins) may reduce CIAKI incidence, several RCTs have evaluated the potential benefit of statin in prevention of CIAKI. [8], [9]Statin's postulated mechanism of kidney protection was through its pleotropic effects, i.e. antioxidant, anti-inflammatory, and antithrombotic actions. However, these previous RCTs and meta-analysis of high-dose statin pre-treatment showed disappointing results. [10]–[12] Recently, three RCTs with relatively large sample size (NAPLES II, PRATO-ACS, TRACK-D trial) have reported promising results favoring prophylactic efficacy of high-dose statin in prevention of CIAKI. [13]–[15] Considering insufficient evidences regarding efficacy of high-dose statin pre-treatment and prognostic importance of CIAKI, we therefore performed a systematic review and comprehensive meta-analysis of all published randomized control trials, in order to evaluate the efficacy of high-dose statin pre-treatment to reduce the incidence of CIAKI in various clinical situations including overall population, chronic kidney disease, or acute coronary syndrome.

## Methods

### Data Sources and Searches

Relevant published or unpublished studies were independently searched in PubMed, Cochrane Central Register of Controlled Trials, EMBASE, the United States National Institutes of Health registry of clinical trials (www.clinicaltrials.gov), and relevant websites (www.crtonline.org, www.clinicaltrialresults.com, www.tctmd.com, www.cardiosource.com, and www.pcronline.com) were also searched. Detailed search strategy was presented in the Method S1. The electronic search strategy was complemented by manual review of reference lists of included articles. References of recent reviews, editorials, and meta-analyses were also examined. No restrictions were imposed on language, study period, or sample size.

### Study Selection

We included RCTs assessing preventive strategies for CIAKI that met following criteria. First, we selected studies which enrolled adult patients undergoing CAG with or without percutaneous coronary intervention (PCI). Second, the intervention was high-dose statin (defined as a daily dose of at least 40 mg of Atorvastatin or equivalent dose of available statins including Simvastatin, Pitavastatin, Fluvastatin, Lovastatin, Pravastatin, or Rosuvastatin), compared with low-dose statin (defined as a daily dose of less than 40 mg of Atorvastatin or equivalent dose of available statins), placebo or none of medication pre-treatment. In cases where a concomitant prophylactic measures were used (for example, NAC, sodium bicarbonate, or other preventive medications), both arms must have shared the same concomitant prophylactic measures, with only a difference in statin protocol. Finally, the incidences of post-procedural CIAKI were reported in both arms, regardless of its definition or the timing of data collection. We excluded RCTs conducted on pediatric patients (including neonates and preterm infants) and randomized crossover trials that assigned patients to both high-dose and low-dose or placebo arms.

### Data Extraction and Quality Assessment

Data extraction and quality assessment was performed as previously described. [16] Summarized data as reported in the published manuscripts were used in the analysis. A standardized form was used to extract characteristics of trials, study design (including randomization sequence generation, allocation concealment, crossover between assigned groups, number of post-randomization withdrawals or follow-up loss), number of study patients, age, eligibility criteria of each trials, definition of CIAKI in each trials, baseline serum creatinine and estimated glomerular filtration rates (eGFR), mean change of serum creatinine after procedure, total cumulative dose of statin before procedure, protocols for statin treatment, hydration protocols, type or mean dosage of radio-contrast agents, the proportion of diabetes mellitus, hypertension, chronic kidney disease, timing of data collection, length of follow-up, adverse events data associated with statin treatment reported on an intention-to-treat basis. We primarily focused our analysis on the effect of prophylactic treatment with high-dose statin on the incidence of CIAKI, not on the surrogate markers of inflammation or oxidative stress. The quality of eligible RCTs was assessed using the Cochrane Collaboration's tool for assessing the risk of bias for RCTs (Table S1 in File S1). Because most previous meta-analyses have reported the methodological quality of each trial using the Jadad score, we also provided this score, as well as the Cochran Collaboration's tool, for each RCT. [17] Two investigators (JML and JP) independently performed screening of titles and abstracts, identified duplicates, reviewed full articles, and determined their eligibility. Disagreements were resolved by discussions. The last search was performed in February 2014.

### Outcomes and Definitions

The primary outcome was the incidence of post-procedural CIAKI within 2–5 days after index procedure. Secondary outcomes included the incidence of post-procedural CIAKI, stratified according to the various subgroups for example, type of contrast agents (iso-osmolar or low-osmolar) used, mean age of the study patients, presence of underlying chronic kidney disease, patients with acute coronary syndrome, NAC usage, or placebo control. All of the patients and outcomes were analyzed according to the originally assigned group.

### Data Synthesis and Analysis

Data synthesis and analsysis was performed as described in detail previously [16], The primary outcome was analyzed by both a random effects model and a fixed effects model. Relative risks (RR) with 95% confidence interval (CI) were presented as summary statistics. The pooled RR was calculated with the DerSimonian and Laird method for random effects model, as well as the Mantel–Haenszel method for fixed effects model. [18] To evaluate the effect of progressive chronological change in study design, such as study population, protocol of statin pre-treatment, hydration protocols or concomitant prophylactic medications including NAC or sodium bicarbonate, we evaluated the impact of publication date on the overall effect of pooled RRs for incidence of CIAKI by a cumulative meta-analysis. Stratified subgroup analyses were performed to assess treatment effects according to the control group (low-dose statin, placebo, or no medication), type of contrast agent, mean age of the patients, underlying chronic kidney disease, acute coronary syndrome, and usage of NAC along with tests for interaction derived from random effects meta-regression. Statistical heterogeneity was assessed by Cochran's Q via a χ2 test and was quantified with the I^2^ test. [19] Publication bias was assessed by funnel plot asymmetry, along with Egger's and Begg's test. The κ statistic was used to assess agreement between investigators for study selection. Results were considered statistically significant at 2-sided p<0.05. Statistical analysis was performed with the use of STATA/SE 12.0 (Stata Corp LP, College Station, Texas, USA). The present study was performed in compliance with the Preferred Reporting Items for Systematic Reviews and Meta-Analyses (PRISMA) guidelines and the review protocol has not been registered (Checklist S1). [20]

## Results

### Search Results

**Figure 1:**
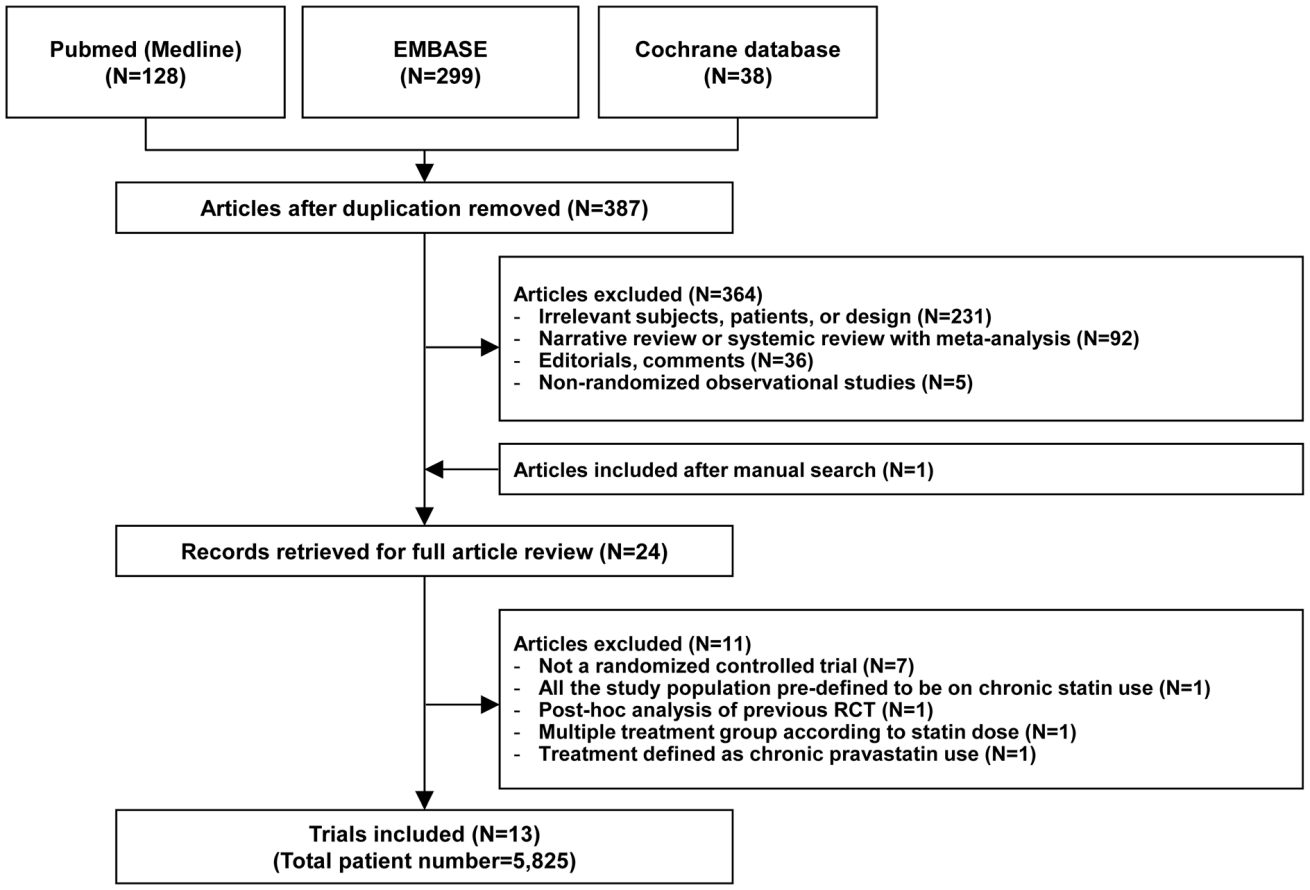
Flow diagram of trial selection. Abbreviations: RCT, randomized controlled trial.

We identified 465 citations from searches as previously described. Among these, 24 studies were retrieved for detailed evaluation, of which 13 RCTs met inclusion criteria (Figure 1). [11]–[15], [21]–[28] These 13 RCTs included a total of 5,825 adult patients, 2,889 (49.6%) of which were allocated to the high-dose statin group and 2,936 (50.4%) of which were allocated to the control group (low-dose statin or placebo group). The characteristics of 11 excluded studies after full article review are summarized in the Method S2. The inter-observer agreement for study selection was high (κ = 0.92).

### Trial Characteristics

The main characteristics of the individual studies are summarized in Table 1 and 2. All trials reported the incidence of CIAKI within 2-5days from index procedure using contrast agents. Four trials exclusively enrolled the patients with chronic kidney disease, which was defined as eGFR of less than 60 ml/min/1.73 m^2^ in PROMISS, Toso et al, and NAPLES II trial, and eGFR of between 30–90 ml/min/1.73 m^2^ in TRACK-D trial. [11]–[14] Only one trial (TRACK-D) exclusively enrolled type 2 diabetes mellitus patients, whereas the others enrolled the patients regardless of diabetes mellitus. [14] Among the 13 trials, 4 trials compared high-dose statin versus low-dose statin pre-treatment. [21], [22], [25], [27] Majority of trials used Atorvastin, whereas 2 trials [11], [21] used Simvastatin, and 2 trials [14], [15] used Rosuvastatin. Total cumulative dose of statin in high-dose statin group ranged from 40 mg to 560 mg of Atorvastatin equivalent dose from 1 to 7 days before CAG. The detailed medication protocols in each included trials are summarized in Table 1. The definition of CIAKI slightly differed across trials. Ten trials [11], [14], [15], [21], [22], [24]–[28] used an increase in serum creatinine of ≥0.5mg/dL or ≥25% from baseline within 48–72 hours after radiocontrast exposure, whereas 2 trials [12], [23] regarded an absolute increase in serum creatinine of ≥0.5 mg/dL within 5 days as their primary definition of CIAKI. One trial (NAPLES II) used an increase in serum cystatin C ≥10% from baseline, which was used in this analysis, although they reported the incidence of CIAKI on the base of the change in serum creatinine as secondary outcomes. [13] All trials evaluated patients with coronary artery disease undergoing CAG with or without percutaneous coronary intervention. Among the trials, 5 studies [15], [21], [26]–[28] exclusively enrolled the patients with acute coronary syndrome including unstable angina and non ST-segment elevation myocardial infarction, and 2 of these 5 studies [21], [28] further included the patients with ST-segment elevation myocardial infarction. Four trials [12], [13], [15], [24] used NAC (600 mg or 1200 mg twice daily) as additional preventive measure of CIAKI in both arms, and 1 trial [13] used sodium bicarbonate solution as primary hydration protocol.

**Table 1:** Characteristics of the study, trial characteristics and protocols.

Trial (Year)	Patients number		Inclusion criteria	Definition of CIN	Medication Protocols		Contrast agent	Contrast volumes (mean), ml		Hydration protocols
	Statin (N = 2889)	Control (N = 2936)			Statin	Control		Statin	Control	
PROMISS (2008)	118	118	CKD patients undergoing CAG or PCI, CrCl≤60 mL/min or SCr≥1.1 mg/dL	Increase of SCr≥0.5 mg/dL or ≥25% at 48 hours	Simvastatin 40 mg bid, 1 day pre-procedure and 1 day post-procedure	Placebo	Visipaque (iodixanol)	173.3	190.9	NS 1 mg/kg/h for 12 h before and 12 h after procedure
Toso et al. (2009)	152	152	CKD patients undergoing CAG or PCI, CrCl<60 mL/min	Increase of SCr≥0.5 mg/dl within 5 days.	Atorvastatin 80 mg/day 2 days pre-procedure and 2 days post-procedure, NAC 1200 mg bid from 1 day before to 1 day post-procedure	Placebo + NAC 1200 mg bid from 1day before to 1 day post-procedure	Visipaque (iodixanol)	151.0	164.0	NS 1 ml/kg/h for 12 h before and after the procedure
Xinwei et al. (2009)	113	115	ACS (UA/NSTEMI) including STEMI patients undergoing PCI	Increase of SCr≥0.5 mg/dL or ≥25% at 48 hours	Simvastatin 80 mg/day from admission to the day before, 20 mg/day after procedure	Simvastatin, 20 mg/day from admission to the end	Visipaque (iodixanol) for CKD, Omnipaque (iohexol) for non-CKD	227.0	240.0	NS 1 ml/kg/h for 6 to 12 h before and 12 h after procedure
Zhou Xia et al. (2009)	50	50	Patients undergoing CAG or PCI	Increase of SCr≥0.5 mg/dL or ≥25% at 72 hours	Atorvastatin 80 mg/day before for 1day,10 mg/day for 6days after procedure	Atorvastatin 10 mg/day for 7 days	Iopamidol 370 mg/ml	118.7	112.9	NS 1000 mL infusion, for 12 h before and 12 h after intervention
Acikel et al. (2010)	80	80	Patients undergoing elective CAG or PCI (excluding ACS), LDL≥70 mg/dl, eGFR≥60 ml/min/1.73 m^2^	Increase of SCr≥0.5 mg/dL at 48 hours	Atorvastatin 40 mg/day 3 days pre-procedure and 2 days post-procedure	None	Omnipaque(iohexol)	105.0	103.0	NS 1 ml/kg/h starting 4 h before and continuing until 24 h after procedure
Ozhan et al. (2010)	60	70	Patients undergoing CAG or PCI, eGFR≥70 ml/min/1.73 m^2^ or SCr≤1.5 mg/dL	Increase of SCr≥0.5 mg/dL or ≥25% at 48 hours	Atorvastatin 80 mg 1 day pre-procedure and 2 days post-procedure, NAC 600 mg bid pre-procedure	No statin pre-procedure, NAC 600 mg bid pre-procedure	Iopamidol	97.0	93.0	NS 1000 ml infusion during 6 h after procedure
Hua et al. (2010)	76	97	Patients undergoing CAG or PCI	Increase of SCr≥0.5 mg/dL or ≥25% at 72 hours	Atorvastatin 80 mg/day pre-procedure	Atorvastatin 20 mg/day pre-procedure	Iopromide	173.0	177.0	NR
ARMYDA-CIN (2011)	120	121	ACS (UA/NSTEMI) Patients undergoing CAG or PCI (excluding high-risk NSTEMI requiring emergency PCI), SCr≤3 mg/dl	Increase of SCr≥0.5 mg/dL or ≥25% at 48 hours	Atorvastatin 80 mg (12 h before) → 40 mg (2 h before), 40 mg for 2days after procedure	Placebo before procedure → Atorvastatin 40 mg for 2days after procedure	Xenetix (iobitridol)	209.0	213.0	For patients CrCl <60 ml/min, NS 1 ml/kg/h for 12 h before and 24 h after intervention
Wei Li et al. (2012)	78	83	STEMI patients undergoing emergency PCI within 12 hours of symptom onset	Increase of SCr≥0.5 mg/dL or ≥25% at 72 hours	Atorvastatin 80 mg loading pre-procedure, long-term 40 mg/day after procedure	Placebo 801mg loading pre-procedure, long-term 40 mg/day after procedure	Ultravist 370 (iopromide)	100.0	103.6	NS 1 ml/kg/h before the procedure and for 12 h after the procedure
NAPLES II (2012)	202	208	CKD patients undergoing CAG or PCI, eGFR<60 ml/min/1.73 m^2^	Increase of Serum Cystatin C concentration ≥10% at 24 hours	Atorvastatin 80 mg before procedure, NAC 1200 mg bid the day before and the day of procedure	No statin pre-procedure, NAC 1200 mg bid the day before and the day of procedure	Visipaque (iodixanol)	177.0	184.0	Sodium bicarbonate solution (154 mEq/L), initial bolus of 3 mL/kg/h for 1 h before procedure, 1 mL/kg/h during and for 6 h after the procedure
CAO et al. (2012)	90	90	Patients undergoing CAG or PCI	Increase of SCr≥0.5 mg/dL or ≥25% at 72 hours	Atorvastatin 40 mg/day from 3days before procedure, 20 mg/day after procedure	Atorvastatin 20 mg/day from 3days before procedure, 20 mg/day after procedure	NR	162.3	158.9	NR
PRATO-ACS (2014)	252	252	ACS (UA/NSTEMI) patients undergoing CAG or PCI (excluding STEMI and high-risk NSTEMI requiring emergency PCI), SCr≤3 mg/dl	Increase of SCr≥0.5 mg/dL or ≥25% at 72 hours	Rosuvastatin 40 mg loading → 20 mg/day before procedure, Rosuvastatin 20 mg/day continued after procedure, NAC 1200 mg bid the day before and the day of procedure	No statin pre-procedure, Atorvastatin 40 mg/day after procedure, NAC 1200 mg bid the day before and the day of procedure	Visipaque (iodixanol)	149.7	138.2	NS 1 ml/kg/h for 12 h both before and after the procedure. Hydration rate was reduced to 0.5 ml/kg/h inboth arms for patients with LVEF <40%
TRACK-D (2014)	1498	1500	Stage 2 or 3 CKD and type II DM patients undergoing CAG or PCI, eGFR ≥30 ml/min/1.73 m^2^ and <90 ml/min/1.73 m^2^ (excluded stage 0,1,4,5 CKD patients)	Increase of SCr≥0.5 mg/dL or ≥25% at 72 hours	Rosuvastatin 10 mg/day from 2 days before to 3 days after procedure → continued after procedure	No statin pre-procedure, Rosuvastatin 10 mg. day 3 days after procedure	Visipaque (iodixanol)	120.0	110.0	NS 1 ml/kg/h started 12 h before and continued for 24 h after procedure

Abbreviations: ACS, acute coronary syndrome; CAG, coronary angiography; CIN, contrast induced nephropathy; CKD, chronic kidney disease; CrCl, creatinine clearance; DM, diabetes mellitus; eGFR, estimated glomerular filtration rate; LDL, low-density lipoprotein; LVEF, left ventricular ejection fraction; NAC, N-acetylcystein; NR, not reported; NS, normal saline (isotonic saline, 0.9%); NSTEMI, non ST-segment elevation myocardial infarction; PCI, percutaneous coronary intervention; SCr, serum creatinine; STEMI, ST-segment elevation myocardial infarction; UA, unstable angina.

**Table 2:** Characteristics of the study, baseline characteristics.

	Mean age, year		Mean baseline SCr (mg/dL)		Mean baseline eGFR (ml/min)					
Trial (Year)	Statin	Control	Statin	Control	Statin	Control	Male proportion	Diabetes Mellitus proportion	Hypertension proportion	Additional measures
PROMISS (2008)	65	66	1.29	1.25	53.46	55.40	72.5%	25.9%	63.2%	None
Toso et al. (2009)	75	76	1.20	1.18	46.00	46.00	64.5%	21.1%	60.5%	NAC
Xinwei et al. (2009)	65	66	0.82	0.83	86.50	93.60	36.0%	20.6%	63.6%	None
Zhou Xia et al. (2009)	60	61	1.04	1.08	76.88	70.54	59.0%	20.0%	75.0%	None
Acikel et al. (2010)	59	61	0.84	0.85	97.70	97.00	63.8%	24.4%	58.1%	None
Ozhan et al. (2010)	54	55	0.88	0.88	92.00	89.00	59.2%	16.2%	22.3%	NAC
Hua et al. (2010)	65	65	1.34	1.40	68.20	66.70	67.1%	26.6%	74.6%	None
ARMYDA-CIN (2011)	65	66	1.04	1.04	79.80	77.00	77.6%	28.2%	75.1%	None
Wei Li et al. (2012)	66	65	0.93	0.93	NR	NR	75.8%	28.0%	80.7%	None
NAPLES II (2012)	70	70	1.32	1.29	42.00	43.00	54.4%	41.2%	86.3%	NAC, bicarbonate
CAO et al. (2012)	63	63	0.85	0.83	109.60	106.80	57.2%	21.7%	37.2%	None
PRATO-ACS (2014)	66	66	0.95	0.96	69.90	69.30	65.7%	21.2%	55.8%	NAC
TRACK-D (2014)	62	61	1.08	1.07	74.16	74.43	65.2%	100.0%	71.9%	None

Abbreviations: ACS, acute coronary syndrome; CAG, coronary angiography; CIN, contrast induced nephropathy; CKD, chronic kidney disease; CrCl, creatinine clearance; DM, diabetes mellitus; eGFR, estimated glomerular filtration rate; LDL, low-density lipoprotein; LVEF, left ventricular ejection fraction; NAC, N-acetylcystein; NR, not reported; NS, normal saline (isotonic saline, 0.9%); NSTEMI, non ST-segment elevation myocardial infarction; PCI, percutaneous coronary intervention; SCr, serum creatinine; STEMI, ST-segment elevation myocardial infarction; UA, unstable angina.

### Risk of Bias within Trials

Figure S1 in File S1 shows the risk of bias graph illustrating the proportion of studies with each of the judgments (‘Yes’, ‘No’, ‘Unclear’) for each entry in the Cochrane Collaboration's tool. A full description of the summary of risk of bias judgments of each study is available in Figure S2 and Table S1 in File S1. All of the included trials were RCTs and no substantial risk of bias was observed in random sequence generation. The included trials showed relatively high methodological quality. Among the 13 RCTs, 5 trials [11], [12], [15], [26], [28] had double-blinded design, whereas others were open-label or non-blinded trials. However, all trials used objective findings (serum creatinine or cystatin C) to define the primary endpoint (the incidence of CIAKI), the authors, therefore, judged that the outcomes were not likely to be influenced by lack of blinding.

### Effect of Statin on the Incidence of Contrast-Induced Acute Kidney Injury

**Figure 2:**
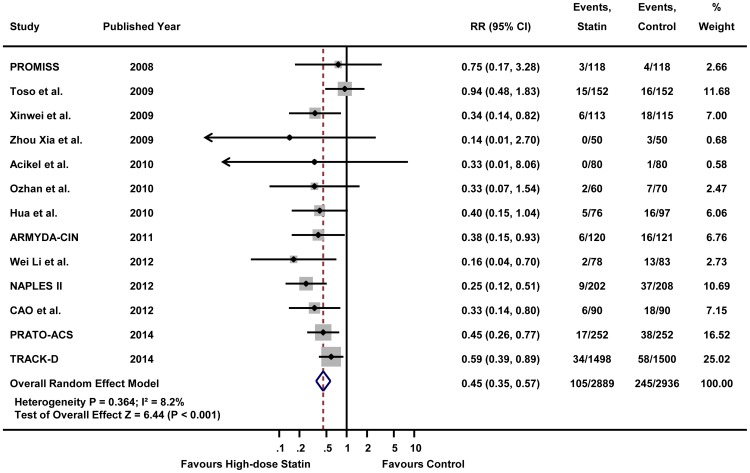
The effect of high-dose statin on the incidence of contrast-induced acute kidney injury by random effects model. Forest plot with relative risks for the incidence of contrast-induced acute kidney injury associated with high-dose statin pre-treatment, compared with control group (low-dose statin or placebo) for individual trials and the pooled population. Abbreviations: CI, confidence intervals; RR, relative risks.

**Figure 3:**
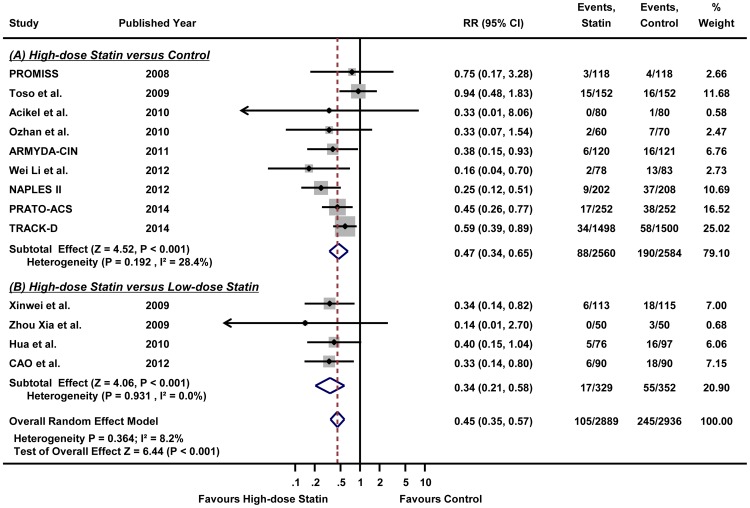
The effect of high-dose statin on the incidence of contrast-induced acute kidney injury, stratified according to the high-dose versus low-dose statin or high-dose versus placebo. Forest plot with relative risks for the incidence of contrast-induced acute kidney injury associated with (A) high-dose statin versus low-dose statin or (B) high-dose statin versus placebo for individual trials and the pooled population. Abbreviations: CI, confidence intervals; RR, relative risks.

As shown in Figure 1, this meta-analysis included 13 RCTs [11]–[15], [21]–[28], all of which provided the incidence of CIAKI. Figure 2 illustrates the RRs of individual study and pooled RR in regards to the incidence of CIAKI, the primary outcome. The overall incidence of CIAKI in the intention-to-treat population was 3.6% (105/2889) in high-dose statin group and 8.3% (245/2936) in control group, respectively. In pooled analysis using random effects model, patients receiving high-dose statin pre-treatment had 55% less risk of CIAKI compared with the control group (RR 0.45, 95% CI 0.35–0.57, p<0.001) (Figure 2). A fixed effects model yielded a similar result (RR 0.44, 95% CI 0.35–0.55, p<0.001) (Figure S3 in File S1). The number needed to treat (NNT) of high-dose statin was 16 in random effects model which means that treatment of 16 patients with high-dose statin will reduce 1 event of CIAKI. There was no significant heterogeneity in either the random effects or the fixed effects model (I^2^ = 8.2%, heterogeneity p = 0.364 for both random and fixed effects model). Since 4 trials compared high-dose versus low-dose statin group and 9 trials compared high-dose versus placebo or no treatment, we performed stratified analysis according to the type of treatment (Figure 3). High-dose statin significantly reduced the risk of CIAKI by 53% (RR 0.47, 95% CI 0.34–0.65, p<0.001, I^2^ = 28.4%, heterogeneity p = 0.192) or 66% (RR 0.34, 95% CI 0.21–0.58, p<0.001, I^2^ = 0.0%, heterogeneity p = 0.931), when compared with placebo or low-dose statin group, respectively.

Visual estimation of the funnel plot indicated no apparent publication or small study effect bias with the support of the Egger's test (p = 0.128) and Begg's test (p = 0.625) (Figure S4 in File S1). No individual study unduly influenced the pooled estimate of high-dose statin for the incidence of CIAKI (Figure S5 in File S1). Cumulative meta-analysis, which sorts trials chronologically, showed no apparent progressive shift of pooled estimate of high-dose statin from a negative to a positive effect, despite of differences in practice patterns or patient populations from 2008 to 2014 (Figure S6 in File S1). Along with the significantly reduced incidence of CIAKI in high-dose statin group, mean change of post-procedural serum creatinine was also significantly lower in the high-dose statin group, compared with control group (SMD −0.37, 95% CI −0.59 to −0.15, p = 0.001) (Figure S7 in File S1).

### Subgroup Analysis

**Figure 4:**
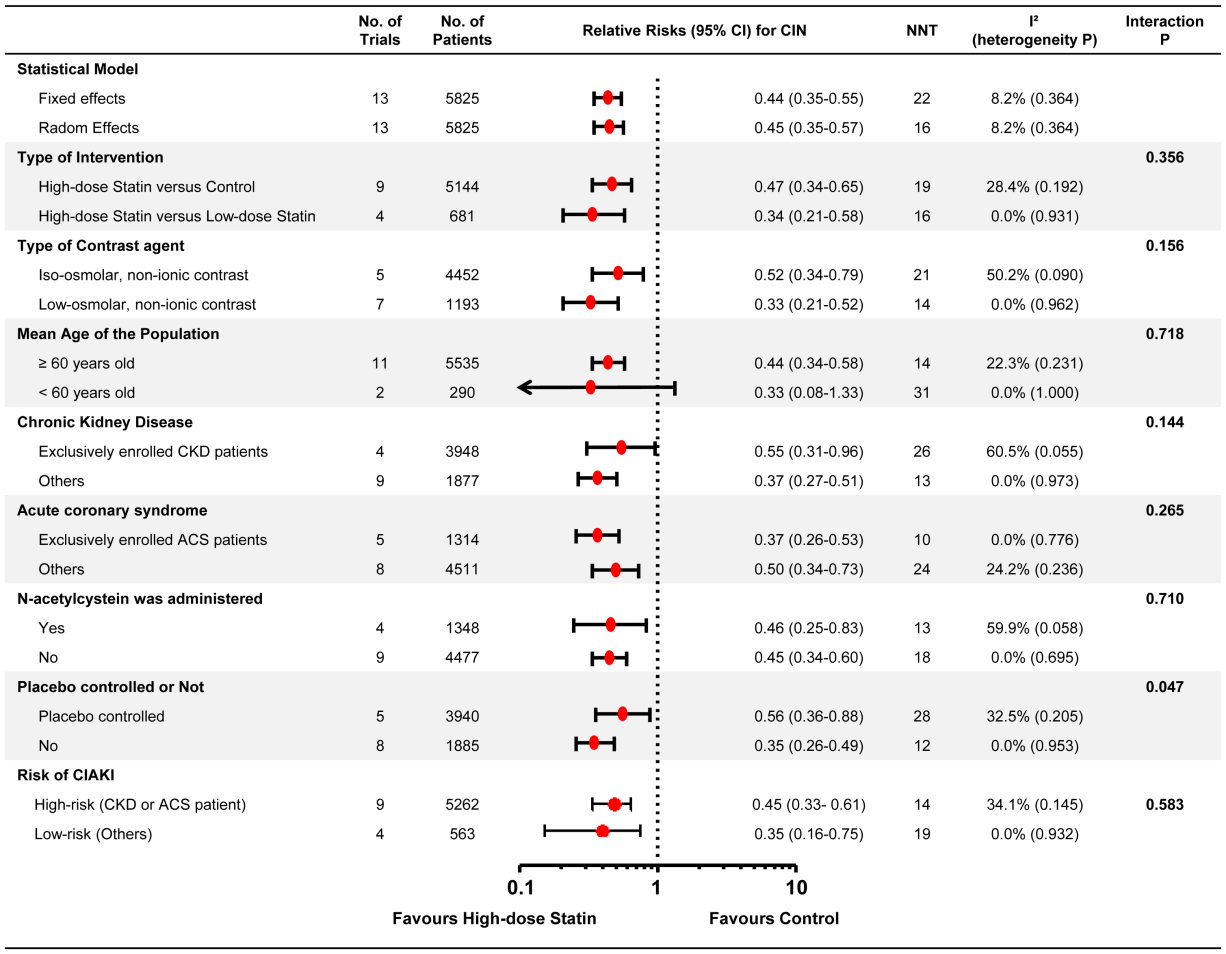
Subgroup analyses according to the study protocols. The forest plot shows relative risks (by random effects model) for the incidence of contrast-induced acute kidney injury associated with high-dose statin pre-treatment, compared with control group (low-dose statin or placebo), stratified according to (1) type of intervention, (2) type of contrast agent, (3) mean age of the patients, (4) underlying chronic kidney disease, (5) acute coronary syndrome, (6) N-acetylcystein as concomitant prophylactic measure, and (7) placebo controlled trial or not. Abbreviations: ACS, acute coronary syndrome; CI, confidence intervals; CKD, chronic kidney disease; RR, relative risks.

The results of subgroup analysis are presented in Figure 4. The beneficial effect of high-dose statin pre-treatment was consistent across all the subgroups, except the subgroup of age less than 60 years old. The high-dose statin showed significantly less development of CIAKI in the patients with old age (≥60 years old), underlying chronic kidney disease, or acute coronary syndrome. When the high-risk subgroup was defined with the patients with chronic kidney disease or acute coronary syndrome, the high-dose statin showed also significant beneficial effect in reducing CIAKI in both high-risk and low-risk subgroup. In addition, the protective effect of high-dose statin was also significant regardless of the osmolality of the contrast agents (iso- or low-osmolar) or concomitant treatment of NAC. Lastly, high-dose statin significantly reduced the incidence of CIAKI compared with placebo or low-dose statin. The NNT of high-dose statin ranged from 12 to 26 (Figure 4). Detailed results of pooled analysis in each subgroup are summarized in the Figure S8-S12 in File S1.

## Discussion

The results of this meta-analysis indicate that high-dose statin pre-treatment in patients undergoing CAG with or without PCI significantly reduced the incidence of CIAKI, compared with control (placebo or low-dose statin). The beneficial effect of high-dose statin was obvious in various subgroups of patients including underlying chronic kidney disease, acute coronary syndrome, or old age (≥60 years). The effect of high-dose statin was also clear regardless of type of contrast agent or concomitant treatment of NAC.

This study is the most up-to-date comprehensive meta-analysis with improved statistical power to address the effect of statin for CIAKI prevention in CAG. [10], [29]–[33] The inconclusive results of previous meta-analyses regarding the efficacy of statin pre-treatment, might mainly originate from the limited sample size of included trials. [29]–[31] Some of these studies included both randomized and non-randomized clinical trials, which might have led to potential bias. [10], [32] In the most recent meta-analysis by Li et al. [33], the authors showed significant benefit of statin pre-treatment in reducing the incidence of CIAKI. However, they argued that statin pre-treatment had no protective effect in the patients with underlying chronic kidney disease (RR 0.79, 95% CI 0.47–1.32, p = 0.37), however, the included studies in this subgroup analysis were only 3 studies with total sample size of 390 in high-dose statin versus 391 in control group. [33] In the present meta-analysis we evaluated over 5,800 patients from 13 RCTs, the benefit of high-dose statin was consistently observed in both overall population and various subgroups including patients with chronic kidney disease (RR 0.55, 95% CI 0.31–0.96, p = 0.036). The limited sample size of the pooled analysis and larger chance of type II error would explain the negative result of Li et al.

Previous studies have suggested that statin protects CIAKI through its pleotropic effect rather than its lipid lowering effect. The pleotropic effect includes enhancement of nitric oxide production, anti-inflammatory, and antioxidative effect. [34], [35] These pleotropic effects could decrease renal cell injury after iodinated contrast exposure. In the NAPLES II trial, high-dose atorvastatin reduced contrast-induced JNK activation and p53 phosphorylation which is the key steps of oxidative stress induced intrinsic apoptosis. [13] Also, statin may modulate the kidney hypoperfusion after radio-contrast exposure by down-regulation of angiotensin receptors and by decrease of endothelin-1 synthesis. [36] Lastly, anti-inflammatory effect of statin may prevent renal cell damage through decrease of pro-inflammatory cytokines which induce tissue factor expression by macrophage and activate nuclear factor-kappa B [37].

Although high-dose statin showed clear beneficial effect in preventing CIAKI, the risk of high-dose statin should be considered. Among the 13 RCTs, only 2 trials reported adverse events related with high-dose statin treatment. [14], [28] Wei Li et al. reported that the rates of hepatotoxicity (defined as>3 times of upper normal limits of alanine aminotransferase within 1 month of the procedure) were 3.85% in high-dose statin group and 1.20% in control group (p = 0.57). In TRACK-D trial, they described that the rates of muscle pain, liver function abnormality, gastrointestinal disorders, edema or rash were not statistically different between high-dose statin and control group without presentation of actual numbers of the complications. Since limited data of adverse events in the included trials, the hazard of high-dose statin pre-treatment could not be evaluated in this meta-analysis. Previous meta-analysis of 35 RCTs comparing statin versus placebo, which was not a meta-analysis for CIAKI, reported that the absolute risk differences (RD) of most frequent adverse drug reactions were as follows; transaminase elevation (RD 4.2%, 95% CI 1.5 to 6.9%), myalgia (RD 2.7%, 95% CI −3.2 to 8.7%), rhabdomyolysis (RD 0.4%, 95% CI −0.1 to 0.9%), and discontinuation due to any adverse drug reaction (RD −0.5%, 95% CI −4.3 to 3.3%). [38] According to this report, the number needed to harm of statin treatment regarding adverse drug reaction are from 24 (hepatotoxicity) to 250 (rhabdomyolysis, defined as creatinine kinase elevations ≥10 times upper normal limit). Considering substantially lower NNT of 16 in this meta-analysis for reducing CIAKI and the clinical importance of CIAKI, high-dose statin pre-treatment before CAG with or without PCI could be considered as an effective prophylactic measure to prevent CIAKI.

## Limitations

Several important limitations of the study should not be ignored. First, this meta-analysis included clinically- and methodologically-diverse studies. Although we included only RCTs to the final analysis and assured statistically insignificant heterogeneity, there were some differences in the enrollment criteria (some studies exclusively enrolled patients with chronic kidney disease or diabetes mellitus), definition of the CIAKI, medication or hydration protocols. Also, basically this meta-analysis comprising 13 RCTs inherently shares the limitations of each trial. Second, variations in the type, dose, and duration of statin pretreatment among the included trials might have potential effects to our results, since all statins may not be equivalent to each other in their pleotropic and nephroprotective effects. Finally, as this study was a study-level meta-analysis, individual patient data were not included in the analysis, and therefore, we could not adjust for patient-level confounders.

## Conclusion

High-dose statin pre-treatment significantly reduced the incidence of CIAKI in patients undergoing CAG. Considering prognostic importance of CIAKI and clear beneficial effect of statin in this meta-analysis, high-dose statin pre-treatment may be more actively employed as an effective prophylactic measure to prevent CIAKI.

## Supporting Information

Checklist S1 PRISMA checklist.
(DOCX)

File S1 **Supporting information files.** Method S1, Search Strategy on Medline, EMBASE and Cochran Central. Method S2, Characteristics of the Excluded Study. Table S1, The Cochrane Collaboration's tool for assessing risk of bias. Figure S1, Risk of bias assessment graph. Risk of bias of each included trial was assessed with the Cochrane Collaboration's tool. This ‘risk of bias graph’ illustrates the proportion of studies with each of the judgments for each entry in the tool. Green represents ‘Yes (low risk of bias)’; yellow, ‘Unclear’; red, ‘No (high risk of bias)’. Figure S2, Risk of bias assessment summary. Risk of bias of each included trial was assessed with the Cochrane Collaboration's tool. This ‘risk of bias summary’ figure presents all of the judgments in a cross-tabulation of study by entry. Green represents ‘Yes (low risk of bias)’; yellow, ‘Unclear’; red, ‘No (high risk of bias)’. Figure S3, The effect of High-dose statin on the incidence of contrast-induced nephropathy by fixed effects model. Forest plot with relative risks for the incidence of contrast-induced nephropathy associated with high-dose statin versus low-dose statin or placebo for individual trials and the pooled population. The squares and the horizontal lines indicate the relative risks (by fixed effects model) and the 95% confidence intervals (CI) for each trial included; the size of each square is proportional to the statistical weight of a trial in the frequentist meta-analysis; diamond indicates the effect estimate derived from meta-analysis, with the center indicating the point estimate and the left and the right ends the 95% CI. Abbreviations: CI, confidence intervals; RR, relative risks. Figure S4, The effect of High-dose statin on mean change of post-procedural serum creatinine. Forest plot with standardized mean difference (SMD) for the mean change of post-procedural serum creatinine from the baseline value associated with high-dose statin versus low-dose statin or placebo for individual trials and the pooled population. The squares and the horizontal lines indicate the SMD (by random effects model) and the 95% confidence intervals (CI) for each trial included; the size of each square is proportional to the statistical weight of a trial in the frequentist meta-analysis; diamond indicates the effect estimate derived from meta-analysis, with the center indicating the point estimate and the left and the right ends the 95% CI. Abbreviations: CI, confidence intervals; SD, standard deviation; SMD, standardized mean difference. Figure S5, Funnel plot for evaluation of publication and small study bias. Figure S6, Influence of individual studies Abbreviations: CI, confidence intervals; RR, relative risks. Figure S7, Cumulative Meta-analysis of high-dose statin on the incidence of contrast-induced nephropathy. The first row shows the effect of one study, the second row shows the cumulative pooled estimates based on the two studies, and so on. The squares and the horizontal lines indicate the cumulative relative risks (by random effects model) and the 95% confidence intervals (CI) for each trial included. Abbreviations: CI, confidence intervals; RR, relative risks. Figure S8, The effect of High-dose statin on the incidence of contrast-induced nephropathy, stratified according to the type of contrast. The squares and the horizontal lines indicate the relative risks (by random effects model) and the 95% confidence intervals (CI) for each trial included; the size of each square is proportional to the statistical weight of a trial in the meta-analysis; diamond indicates the effect estimate derived from meta-analysis, with the center indicating the point estimate and the left and the right ends the 95% CI. Abbreviations: CI, confidence intervals; RR, relative risks. Figure S9, The effect of High-dose statin on the incidence of contrast-induced nephropathy, stratified according to the mean age of the patients. The squares and the horizontal lines indicate the relative risks (by random effects model) and the 95% confidence intervals (CI) for each trial included; the size of each square is proportional to the statistical weight of a trial in the meta-analysis; diamond indicates the effect estimate derived from meta-analysis, with the center indicating the point estimate and the left and the right ends the 95% CI. Abbreviations: CI, confidence intervals; RR, relative risks. Figure S10, The effect of High-dose statin on the incidence of contrast-induced nephropathy, stratified according to the underlying chronic kidney disease. The squares and the horizontal lines indicate the relative risks (by random effects model) and the 95% confidence intervals (CI) for each trial included; the size of each square is proportional to the statistical weight of a trial in the meta-analysis; diamond indicates the effect estimate derived from meta-analysis, with the center indicating the point estimate and the left and the right ends the 95% CI. Abbreviations: CI, confidence intervals; CKD, chronic kidney disease; eGFR, estimated glomerular filtration rates; RR, relative risks. Figure S11, The effect of High-dose statin on the incidence of contrast-induced nephropathy, stratified according to the acute coronary syndrome. The squares and the horizontal lines indicate the relative risks (by random effects model) and the 95% confidence intervals (CI) for each trial included; the size of each square is proportional to the statistical weight of a trial in the meta-analysis; diamond indicates the effect estimate derived from meta-analysis, with the center indicating the point estimate and the left and the right ends the 95% CI. Abbreviations: CI, confidence intervals; RR, relative risks. Figure S12, The effect of High-dose statin on the incidence of contrast-induced nephropathy, stratified according to the concomitant treatment of N-acetylcystein. The squares and the horizontal lines indicate the relative risks (by random effects model) and the 95% confidence intervals (CI) for each trial included; the size of each square is proportional to the statistical weight of a trial in the meta-analysis; diamond indicates the effect estimate derived from meta-analysis, with the center indicating the point estimate and the left and the right ends the 95% CI. Abbreviations: CI, confidence intervals; RR, relative risks.
(PDF)
