# Supplementary material for: Efficacy of Short-Term High-Dose Statin Pretreatment in Prevention of Contrast-Induced Acute Kidney Injury: Updated Study-Level Meta-Analysis of 13 Randomized Controlled Trials
Source: PLoS One. 2014 Nov 4;9(11):e111397. doi: 10.1371/journal.pone.0111397 (PMC4219719; doi:10.1371/journal.pone.0111397)

File S1. Supporting Information File

Efficacy of Short-Term High-Dose Statin Pretreatment in Prevention of Contrast-Induced Acute Kidney Injury : Updated Study-Level Meta-Analysis of 13 Randomized Controlled Trials

Joo Myung Lee, MD, MPH[1†], Jonghanne Park, MD[1†], Ki-Hyun Jeon, MD[1],

Ji-hyun Jung, MD[1], Sang Eun Lee, MD, PhD[1], Jung-Kyu Han, MD, PhD[1], Hack-Lyoung Kim, MD, PhD[2], Han-Mo Yang, MD, PhD[1], Kyung Woo Park, MD, PhD[1], Hyun-Jae Kang, MD, PhD[1], Bon-Kwon Koo, MD, PhD[1], Sang-Ho Jo, MD[3],

Hyo-Soo Kim, MD, PhD[1,4]

[1]Department of Internal Medicine and Cardiovascular Center, Seoul National University Hospital, Seoul, Korea

[2]Cardiovascular Center, Seoul National University Boramae Medical Center, Seoul, Korea

[3]Division of Cardiology, Department of Internal Medicine, Hallym University Sacred Heart Hospital, Anyang-si, Gyeonggi-do, Korea

[4]Department of Molecular Medicine and Biopharmaceutical Sciences, Graduate School of Convergence Science and Technology, Seoul National University, Seoul, Korea.

Supporting Methods

Method S1. Search strategy

Pubmed			EMBASE			Cochrane Library		
#21	**#18** AND **#19** AND **#20**	128	#22	**#21** AND (**'clinical trial'**/de OR **'controlled clinical trial'**/de OR **'randomized controlled trial'**/de)	299	#21	**#18** AND **#19** AND **#20**	38
#20	**#12** OR **#13** OR **#14** OR **#15** OR **#16** OR **#17**	483,790	#21	**#18** AND **#19** AND **#20**	1,133	#20	**#12** OR **#13** OR **#14** OR **#15** OR **#16** OR **#17**	11,822
#19	**#3** OR **#4** OR **#5** OR **#6** OR **#7** OR **#8** OR **#9** OR **#10** OR **#11**	38,965	#20	**#12** OR **#13** OR **#14** OR **#15** OR **#16** OR **#17**	715,000	#19	**#3** OR **#4** OR **#5** OR **#6** OR **#7** OR **#8** OR **#9** OR **#10** OR **#11**	7,380
#18	**#1** OR **#2**	789,756	#19	**#3** OR **#4** OR **#5** OR **#6** OR **#7** OR **#8** OR **#9** OR **#10** OR **#11**	110,188	#18	**#1** OR **#2**	22,825
#17	**'kidney failure'**	79,639	#18	**#1** OR **#2**	1,376,056	#17	**'kidney failure'**:ti,ab,kw	5,096
#16	**'renal insufficiency'**	31,322	#17	**'kidney failure'**/syn	253,346	#16	**'renal insufficiency'**:ti,ab,kw	1,504
#15	**'renal failure'**	75,804	#16	**'renal insufficiency'**/syn	253,346	#15	**'renal failure'**:ti,ab,kw	3,015
#14	**'kidney injury'**	35,238	#15	**'renal failure'**/syn	253,346	#14	**'kidney injury'**:ti,ab,kw	866
#13	**'nephrotoxicity'**	12,899	#14	**'kidney injury'**/syn	39,568	#13	**'nephrotoxicity'**:ti,ab,kw	1,221
#12	**'nephropathy'**	447,935	#13	**'nephrotoxicity'**/syn	55,283	#12	**'nephropathy'**:ti,ab,kw	3,281
#11	**'cerivastatin'**	698	#12	**'nephropathy'**/syn	692,175	#11	**'cerivastatin'**:ti,ab,kw	145
#10	**'lovastatin'**	9,558	#11	**'cerivastatin'**/syn	4,015	#10	**'lovastatin'**:ti,ab,kw	760
#9	**'pravastatin'**	4,078	#10	**'lovastatin'**/syn	13,385	#9	**'pravastatin'**:ti,ab,kw	1,335
#8	**'simvastatin'**	7,670	#9	**'pravastatin'**/syn	16,960	#8	**'simvastatin'**:ti,ab,kw	2,000

#7	**'rosuvastatin'**	2,100	#8	**'simvastatin'**/syn	27,935	#7	**'rosuvastatin'**:ti,ab,kw	724
#6	**'atorvastatin'**	6,201	#7	**'rosuvastatin'**/syn	8,250	#6	**'atorvastatin'**:ti,ab,kw	2,383
#5	**'hydroxymethylglutaryl'**	23,081	#6	**'atorvastatin'**/syn	24,302	#5	**'hydroxymethylglutaryl'**	3279
#4	**'hmg-coa'**	9,392	#5	**'hydroxymethylglutaryl'**	101,523	#4	**'hmg-coa'**:ti,ab,kw	683
#3	**'statin'**	31,933	#4	**'hmgcoa'**/syn	102,934	#3	**'statin'**:ti,ab,kw	2,114
#2	**radiocontrast**	913	#3	**'statin'**/syn	21,670	#2	**radiocontrast**	116
#1	**'contrast'**	789,463	#2	**radiocontrast**	1,175	#1	**'contrast'**:ti,ab,kw	22,804
			#1	**'contrast'**/syn	1,375,901			

Method S2. Characteristics of the Excluded Studies

No.	Title	First Author	Journal	Main Reason for Exclusion
1	Statin therapy reduces contrast-induced nephropathy: an analysis of contemporary percutaneous interventions	Khanal, S et al.	Am J Med 2005	Not a randomized controlled trial
2	Usefulness of statin pretreatment to prevent contrast-induced nephropathy and to improve long-term outcome in patients undergoing percutaneous coronary intervention	Patti, G et al.	Am J Cardiol 2008	Not a randomized controlled trial
3	Association between high sensitivity C-reactive protein and contrast induced acute kidney injury in patients with acute coronary syndrome undergoing percutaneous coronary intervention: impact of atorvastatin	Su, J. Z et al.	Zhonghua Xin Xue Guan Bing Za Zhi 2011	Observational study<br>Multiple treatment group according to statin dose
4	Impact on renal function of rosuvastatin preload prior to elective percutaneous coronary intervention in chronic statin users	de Oliveira, M. S. et al.	Revista Brasileira de Cardiologia Invasiva 2012	All the study population pre-defined to be on chronic statin use
5	Effect of statins in contrast-induced nephropathy after coronary angiography	Selmi, W et al.	JACC: Cardiovascular Interventions 2013	Post-hoc analysis of previeous RCT
6	The potential role of statins in contrast nephropathy	Attallah, N et al	Clin Nephrol 2004	Not a randomized controlled trial
7	Statin therapy and contrast-induced nephropathy after primary angioplasty	Bouzas-Mosquera, A et al.	Int J Cardiol 2009	Not a randomized controlled trial

8	Preventive effect of statin pretreatment on contrast-induced acute kidney injury in patients undergoing coronary angioplasty: Propensity score analysis from a multicenter registry	Hoshi, T et al.	Int J Cardiol 2014	Not a randomized controlled trial
9	Statins for prevention of contrast-induced nephropathy in patients undergoing non-emergent percutaneous coronary intervention	Kandula, P et al.	Nephrology (Carlton) 2010	Not a randomized controlled trial
10	Prevention of contrast-induced nephropathy by chronic pravastatin treatment in patients with cardiovascular disease and renal insufficiency	Yoshida, S et al.	J Cardiol 2009	Not a randomized controlled trial<br>Treatment group defined as chronic pravastatin use
11	Effect of statins on contrast-induced nephropathy in patients with acute myocardial infarction treated with primary angioplasty	Zhao, J. L. et al.	Int J Cardiol 2008	Not a randomized controlled trial

Supporting Tables

Table S1. The Cochrane Collaboration's tool for assessing risk of bias

Study	Domain	Support for judgment & review authors' judgment	Jadad Score
**Acikel et al.**	Random Sequence Generation	Low risk of bias. Patients were randomized in 1:1 ratio to either CG or AG using a simple randomization method.	2
	Allocation concealment	Low risk of bias. Allocation concealment was not maintained througout the study. First patient was randomly assigned to AG via a coin toss. Subsequent patients were then assigned to CG or AG in an alternating manner. However, the review authors judge that the outcome is not likely to be influenced by lack of concealment since all of the clinical outcome were objective findings.	
	Blinding of participants and personnel	Low risk of bias. The study was a open-label trial comparing atrovastatin and non-treatment group. However, the review authors judge that the outcome is not likely to be influenced since the treatmnet protocol was pre-specified and the primary and additional endpoints were laboratory results.	
	Blinding of outcome assessment	Unclear. Insufficient information to permit judgment. Independency of laboratory measurement not stated.	
	Incomplete outcome data	Low risk of bias. A total of 160 patients were completely followed to the end of the study. None of the patients were excluded from final analysis.	
	Selective reporting	Low risk of bias. All of the study's pre-specified (primary and secondary) outcomes that are of interest in the review have been reported in the pre-specified manner.	
	Other sources of bias	Low risk of bias. The study appears to be free of other sources of bias.	
**ARMYDA-CIN**	Random Sequence Generation	Low risk of bias. Patients were assigned to the study arm using an electronic spreadsheet indicating the group assignment by random numbers.	5
	Allocation concealment	Low risk of bias. Central randomization. Randomization blocks were created and distributed to the 2 centers.	
	Blinding of participants and personnel	Low risk of bias. Double blinded placebo controlled clinical trial Physicians performing the procedure and follow-up assessment were not aware of the randomization assignment.	
	Blinding of outcome assessment	Low risk of bias. The primary and secondary ouputs include objective labroatory test.	
	Incomplete outcome data	Low risk of bias. A total of 241 patients were completely followed to the end of the study. None of the patients were excluded from final analysis	
	Selective reporting	Low risk of bias. All of the study's pre-specified (primary and secondary) outcomes that are of interest in the review have been reported in the pre-specified manner	
	Other sources of bias	Low risk of bias. The study appears to be free of other sources of bias.	
**Cao et al.**	Random Sequence Generation	Low risk of bias. Patients were randomly allocated	2
	Allocation concealment	Unclear. Insufficient information to permit judgment, it is not clear whether allocation	

		concealment was maintained throughout the study.	
	Blinding of participants and personnel	Low risk of bias. Open-label study. Although, both patients and investigators were aware of the study group assignment, the review authors judge that the outcome is note likely to be influenced by unblinding since the treatmnet protocol was pre-specified and the primary endpoint was laboratory result.	
	Blinding of outcome assessment	Unclear. Insufficient information to permit judgment. Independency of laboratory measurement and detection method not stated.	
	Incomplete outcome data	Low risk of bias. A total of 180 patients were completely followed to the end of the study. None of the patients were excluded from final analysis.	
	Selective reporting	Low risk of bias. All of the study's pre-specified (primary and secondary) outcomes that are of interest in the review have been reported in the pre-specified manner.	
	Other sources of bias	Low risk of bias. The study appears to be free of other sources of bias.	
**Hua et al.**	Random Sequence Generation	Low risk of bias. Patients were randomly allocated.	2
	Allocation concealment	Unclear. Insufficient information to permit judgment, it is not clear whether allocation concealment was maintained throughout the study.	
	Blinding of participants and personnel	Low risk of bias. Open-label study. Although, both patients and investigators were aware of the study group assignment, the review authors judge that the outcome is note likely to be influenced by unblinding since the treatmnet protocol was pre-specified and the primary endpoint was laboratory result.	
	Blinding of outcome assessment	Unclear. Insufficient information to permit judgment, it is not clear who performed serum creatinine measurement and the laboratory method.	
	Incomplete outcome data	Low risk of bias. A total of 173 patients were completely followed to the end of the study. None of the patients were excluded from final analysis.	
	Selective reporting	Low risk of bias. All of the study's pre-specified (primary and secondary) outcomes that are of interest in the review have been reported in the pre-specified manner	
	Other sources of bias	Low risk of bias. The study appears to be free of other sources of bias.	
**NAPLES II .**	Random Sequence Generation	Low risk of bias. Randomization was performed by a 1:1 ratio with computer-generated random numbers.	3
	Allocation concealment	Unclear. Insufficient information to permit judgment, it is not clear whether allocation concealment was maintained throughout the study.	
	Blinding of participants and personnel	Low risk of bias. Open-label study. Although, both patients and investigators were aware of the study group assignment, the review authors judge that the outcome is note likely to be influenced by unblinding since the treatmnet protocol was pre-specified and the primary and additional endpoints were laboratory results.	
	Blinding of outcome assessment	Unclear. Insufficient information to permit judgment. Independency of laboratory measurement and detection method not stated.	
	Incomplete outcome data	Low risk of bias. Despite of the 4% loss to follow-up rate, the authors provided daate of 17 patients lost at follow-up which were largely similarto those analyzed.	

	Selective reporting	Low risk of bias. All of the study’s pre-specified (primary and secondary) outcomes that are of interest in the review have been reported in the pre-specified manner.	
	Other sources of bias	Low risk of bias. The study appears to be free of other sources of bias.	
**Ozhan et al.**	Random Sequence Generation	Low risk of bias. The patients were randomized to a short-term highdose atorvastatin plus NAC or only NAC.	3
	Allocation concealment	Unclear. Insufficient information to permit judgment, it is not clear whether allocation concealment was maintained throughout the study.	
	Blinding of participants and personnel	Low risk of bias. Open-label study. Although, both patients and investigators were aware of the study group assignment, the review authors judge that the outcome is note likely to be influenced by unblinding since the treatmnet protocol was pre-specified and the primary endpoint was laboratory result.	
	Blinding of outcome assessment	Unclear. Insufficient information to permit judgment. Independency of laboratory measurement not stated.	
	Incomplete outcome data	Low risk of bias. A total of 130 patients were completely followed to the end of the study. None of the patients were excluded from final analysis.	
	Selective reporting	Low risk of bias. All of the study’s pre-specified (primary and secondary) outcomes that are of interest in the review have been reported in the pre-specified manner.	
	Other sources of bias	Low risk of bias. The study appears to be free of other sources of bias.	
**PRATO-ACS**	Random Sequence Generation	Low risk of bias. Randomization was performed by computerized using an electronic spreadsheet with blocks of 50 patients each.	5
	Allocation concealment	Low risk of bias. Randomization was performed on admission by computerized assignment.	
	Blinding of participants and personnel	Low risk of bias. Although, both patients and investigators were aware of the study group assignment, the review authors judge that the outcome is note likely to be influenced by unblinding since the treatmnet protocol was pre-specified and the primary and additional endpoints were laboratory results.	
	Blinding of outcome assessment	Low risk of bias. All tests were done in the author's hospital laboratory with consistent methodology.	
	Incomplete outcome data	Low risk of bias. Data are missing in both intervention groups, but reasons for these are both reported and balanced.	
	Selective reporting	Low risk of bias. All of the study’s pre-specified (primary and secondary) outcomes that are of interest in the review have been reported in the pre-specified manner. The study protocol is available (http://clinicaltrials.gov/show/NCT01185938)	
	Other sources of bias	Low risk of bias. The study appears to be free of other sources of bias.	
**PROMISS**	Random Sequence Generation	Low risk of bias. Patients were randomized 1:1 by computer generated permuted block of 6 patients.	5
	Allocation concealment	Low risk of bias. Patients were randomized by computer generated codes provided by the research memeber of the center after enrollment.	
	Blinding of participants and personnel	Low risk of bias. Eligible patients were randomly assigned to receive simvastatin or placebo.	

		Both patients and investigators were blinded to study group assignment	
	Blinding of outcome assessment	Low risk of bias. Serum creatinine levels were determined in a blinded fashion by laboratory personnel. Analysis was performed using autoanalyzers located in independent department of the center.	
	Incomplete outcome data	Low risk of bias. Data are missing in both intervention groups, but reasons for these are both reported and balanced. (6/124 (4.8%) from treatment group and 5/123 (4.1%) due to incomplete laboratory test results).	
	Selective reporting	Low risk of bias. All of the study's pre-specified (primary and secondary) outcomes that are of interest in the review have been reported in the pre-specified manner. The study protocol is available (http://clinicaltrials.gov/show/NCT00259441)	
	Other sources of bias	Low risk of bias. The study appears to be free of other sources of bias.	
**Toso et al.**	Random Sequence Generation	Low risk of Bias. Randomization was performed by computerized assignment.	5
	Allocation concealment	Unclear. Insufficient information to permit judgment, it is not clear whether allocation concealment was maintained throughout the study, but the review authors judge that the outcome is not likely to be influenced by lack of blinding since all of the clinical outcome were objective findings.	
	Blinding of participants and personnel	Low risk of Bias. Although the study was a open-label placebo controlled trial, the review authors judge that the outcome is note likely to be influenced by unblinding since the treatmnet protocol was pre-specified and the primary and additional endpoints were laboratory results or objective outcome (death and need for dialysus/hemofiltration)	
	Blinding of outcome assessment	Low risk of bias. Serum creatinine levels tests were performed in the same hospital laboratory with consistent methods.	
	Incomplete outcome data	Low risk of bias. All patients presented for creatinine determination and clinical follow-up at 1 month.	
	Selective reporting	Low risk of bias. All of the study's pre-specified (primary and secondary) outcomes that are of interest in the review have been reported in the pre-specified manner.	
	Other sources of bias	Low risk of bias. The study appears to be free of other sources of bias.	
**TRACK-D**	Random Sequence Generation	Low risk of bias. Block randomization was performed using computerized assignment with a block size of 6.	3
	Allocation concealment	Low risk of bias. Randomization was performed using computerized assignment by blinded envelopes.	
	Blinding of participants and personnel	Low risk of bias. Open-label study. Although, both patients and investigators were aware of the study group assignment, the review authors judge that the outcome is note likely to be influenced by unblinding since the treatmnet protocol was pre-specified and the primary endpoint was laboratory result.	
	Blinding of outcome assessment	Low risk of bias. All events and biomarkers were collected and adjudicated by a blinded, independent committee.	

	Incomplete outcome data	Low risk of bias. Data are missing in both intervention groups, but reasons for these are both reported and balanced.<br>The study protocol is available (http://clinicaltrials.gov/show/NCT00786136)	
	Selective reporting	Low risk of bias. All of the study's pre-specified (primary and secondary) outcomes that are of interest in the review have been reported in the pre-specified manner.	
	Other sources of bias	Low risk of bias. The study appears to be free of other sources of bias.	
**Wei Li et al.**	Random Sequence Generation	Low risk of bias. Eligible patients were randomly assigned in a 1: 1 ratio to receive atorvastatin or placebo	4
	Allocation concealment	Unclear. Insufficient information to permit judgment, it is not clear whether allocation concealment was maintained throughout the study, but the review authors judge that the outcome is not likely to be influenced since all of the clinical outcome were objective findings.	
	Blinding of participants and personnel	Low risk of bias. The study is a double-blinded placebo-controlled trial.	
	Blinding of outcome assessment	Low risk of bias. The primary and secondary ouputs include objective labroatory test.	
	Incomplete outcome data	Low risk of bias. A total of 161 patients were completely followed to the end of the study. None of the patients were excluded from final analysis. Exlcusion of non-PCI patient was done after randomization (12/90 (13.3%) for treatment group and 7/90 (7.8%) for control group). However, the review authors judge that the outcome is not likely to be influenced since the baseline characteristics of both group remained similar between groups..	
	Selective reporting	Low risk of bias. All of the study's pre-specified (primary and secondary) outcomes that are of interest in the review have been reported in the pre-specified manner	
	Other sources of bias	Low risk of bias. The study appears to be free of other sources of bias.	
**Xinwei et al.**	Random Sequence Generation	Low risk of bias. Patients were randomized 1:1 by a computer-generated permuted block of 8 patients	3
	Allocation concealment	Low risk of bias. Randomized codes were provided by the research member of the center who was unaware of the data in the present study.	
	Blinding of participants and personnel	Low risk of bias. Although, both patients and investigators were aware of the study group assignment, the review authors judge that the outcome is note likely to be influenced by unblinding since the treatmnet protocol was pre-specified and the primary and additional endpoints were laboratory results.	
	Blinding of outcome assessment	Unclear. Insufficient information to permit judgment. Independency of laboratory measurement and detection method not stated.	
	Incomplete outcome data	Low risk of bias. A total of 228 patients were completely followed to the end of the study. None of the patients were excluded from final analysis.	
	Selective reporting	Low risk of bias. All of the study's pre-specified (primary and secondary) outcomes that are of interest in the review have been reported in the pre-specified manner.	
	Other sources of bias	Low risk of bias. The study appears to be free of other sources of bias.	
**Zhao Xia et al.**	Random Sequence Generation	Low risk of bias. Patients were randomly allocated	3

Allocation concealment	Unclear. Insufficient information to permit judgment, it is not clear whether allocation concealment was maintained throughout the study.
Blinding of participants and personnel	Low risk of Bias. Although the study was a open-label trial, the review authors judge that the outcome is note likely to be influenced by unblinding since the treatmnet protocol was pre-specified and the primary and additional endpoints were laboratory results.
Blinding of outcome assessment	Unclear. Insufficient information to permit judgment, it is not clear who performed serum creatinine measurement and the laboratory method.
Incomplete outcome data	Low risk of bias. A total of 100 patients were completely followed to the end of the study. None of the patients were excluded from final analysis.
Selective reporting	Low risk of bias. All of the study's pre-specified (primary and secondary) outcomes that are of interest in the review have been reported in the pre-specified manner.
Other sources of bias	Low risk of bias. The study appears to be free of other sources of bias.

Figure S1. Risk of Bias Graph and Summary Figure

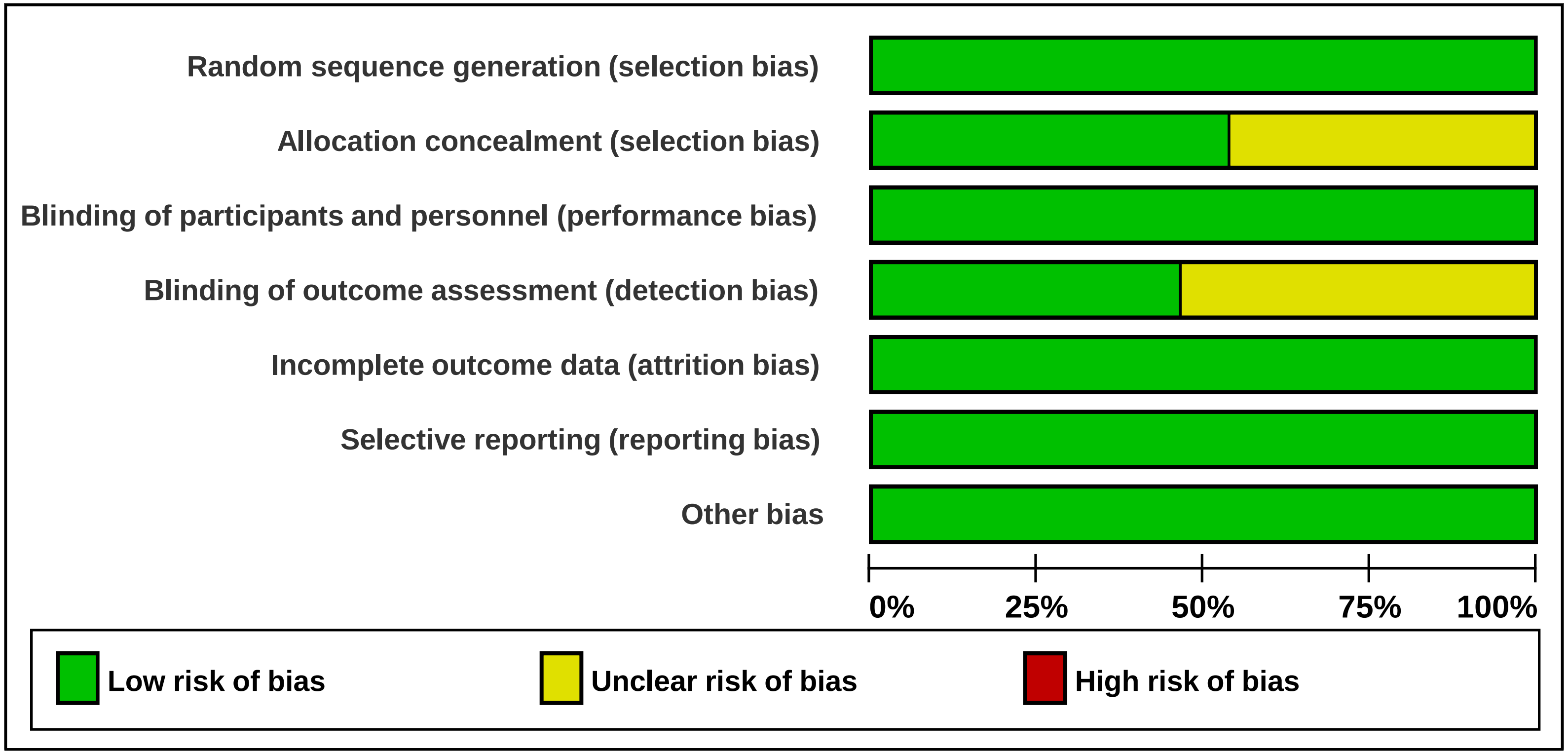

Figure S2. Risk of Bias Graph and Summary Figure

	Zhao Xia et al.	Xinwei et al.	Wei li et al.	TRACK-D	Toso et al.	PROMISS	PRATO-ACS	Ozhan et al.	NAPLES II	Hua et al.	Cao et al.	ARMYDA CIN	Acikel et al.
Random sequence generation (selection bias)	+	+	+	+	+	+	+	+	+	+	+	+	+
Allocation concealment (selection bias)	?	+	+	+	?	+	+	?	?	?	?	+	+
Blinding of participants and personnel (performance bias)	+	+	+	+	+	+	+	+	+	+	+	+	+
Blinding of outcome assessment (detection bias)	?	?	+	+	+	+	+	?	?	?	?	+	?
Incomplete outcome data (attrition bias)	+	+	+	+	+	+	+	+	+	+	+	+	+
Selective reporting (reporting bias)	+	+	+	+	+	+	+	+	+	+	+	+	+
Other bias	+	+	+	+	+	+	+	+	+	+	+	+	+

Figure S3. Overall Fixed Effects Model

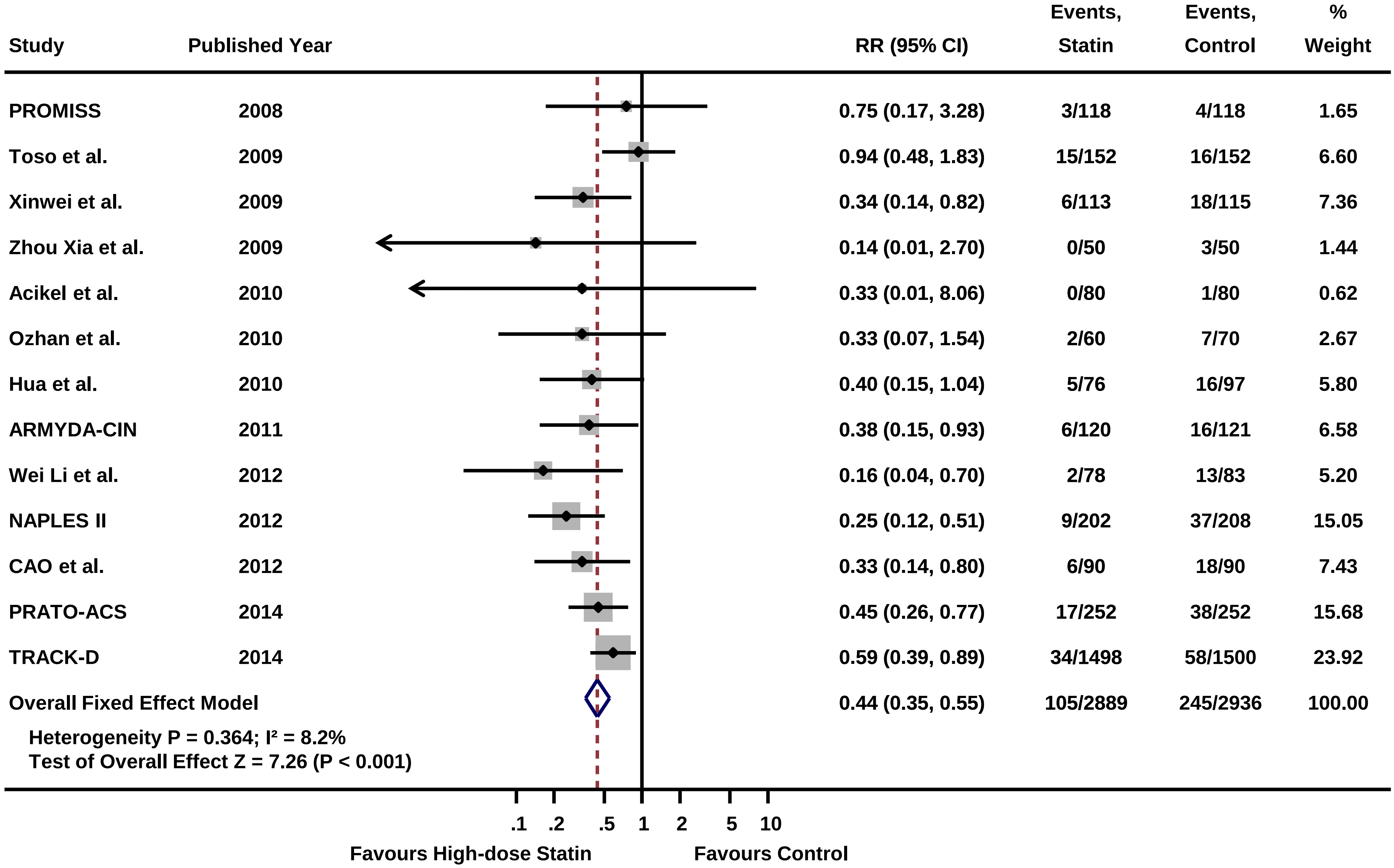

Figure S4. Assessment of the Small Study Effect Bias

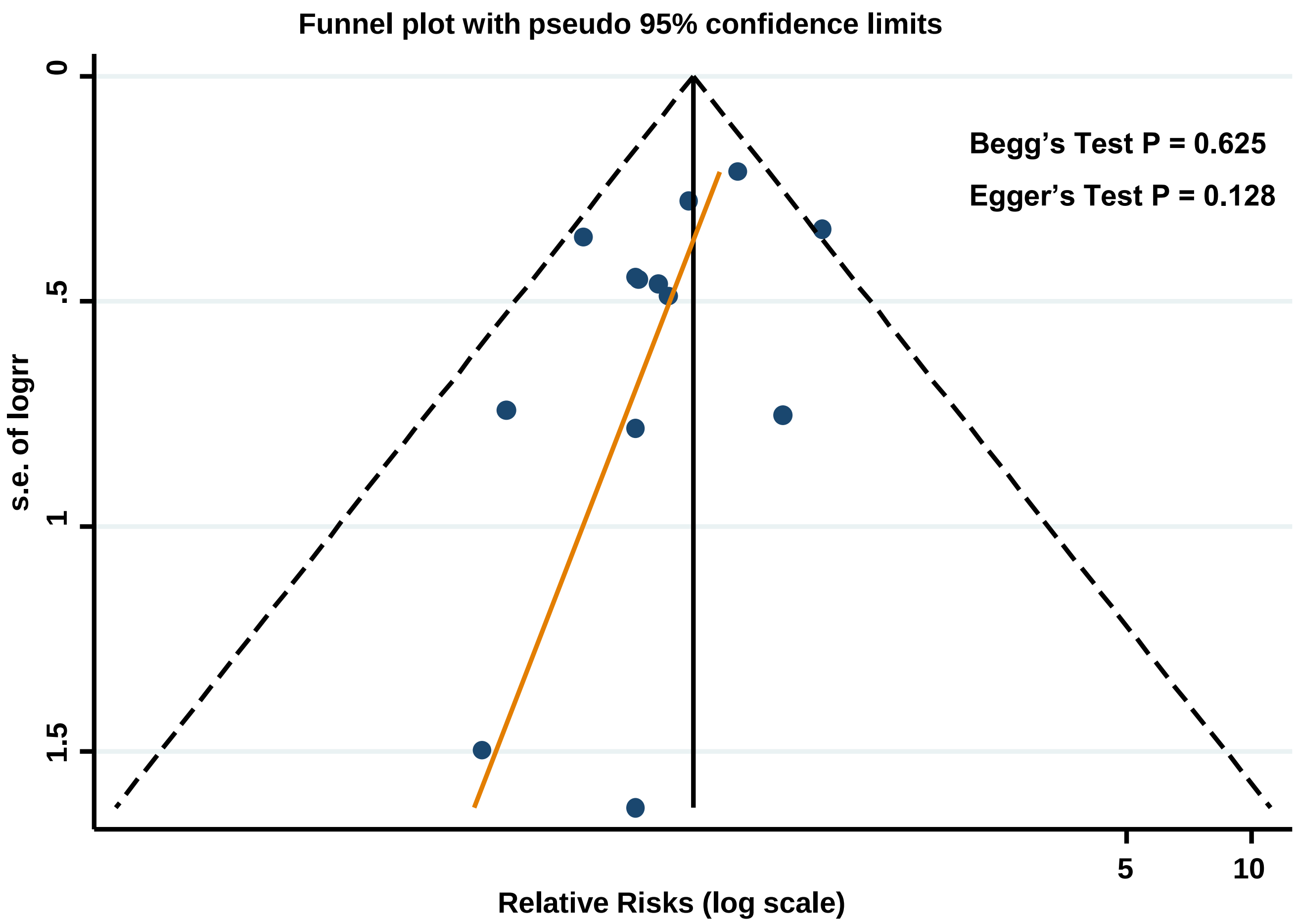

Figure S5. Influence of Individual Studies

	Study Omitted	Year	RR (95% CI)
1	PROMISS	2008	0.44 (0.34, 0.57)
2	Toso et al.	2009	0.42 (0.33, 0.53)
3	Xinwei et al.	2009	0.45 (0.35, 0.59)
4	Zhou Xia et al.	2009	0.45 (0.35, 0.58)
5	Acikel et al.	2010	0.44 (0.34, 0.58)
6	Ozhan et al.	2010	0.45 (0.35, 0.58)
7	Hua et al.	2010	0.45 (0.34, 0.58)
8	ARMYDA-CIN	2011	0.45 (0.34, 0.59)
9	Wei Li et al.	2012	0.47 (0.37, 0.59)
10	NAPLES II	2012	0.49 (0.39, 0.62)
11	CAO et al.	2012	0.46 (0.35, 0.59)
12	PRATO-ACS	2014	0.44 (0.33, 0.59)
13	TRACK-D	2014	0.41 (0.32, 0.54)
Total (Random Effect Model)			0.45 (0.35, 0.57)

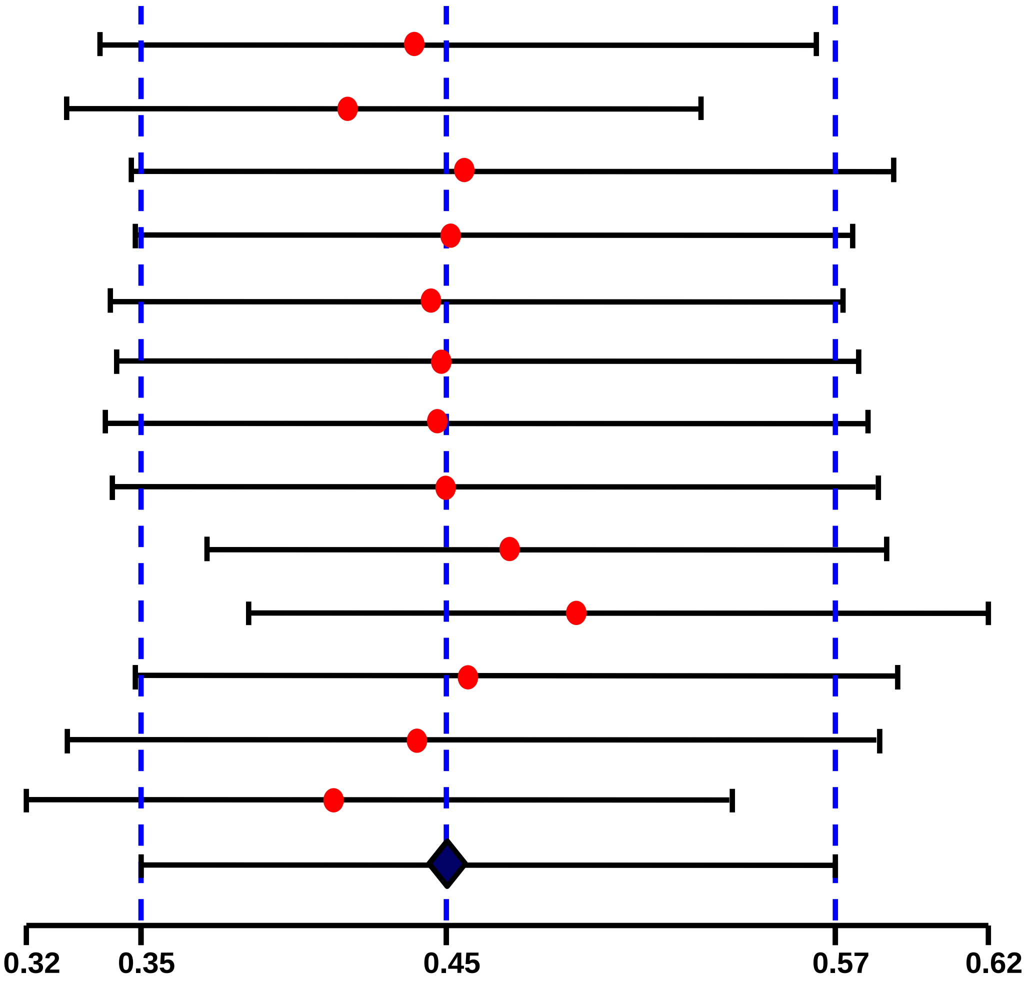

Figure S6. Cumulative Meta-analysis

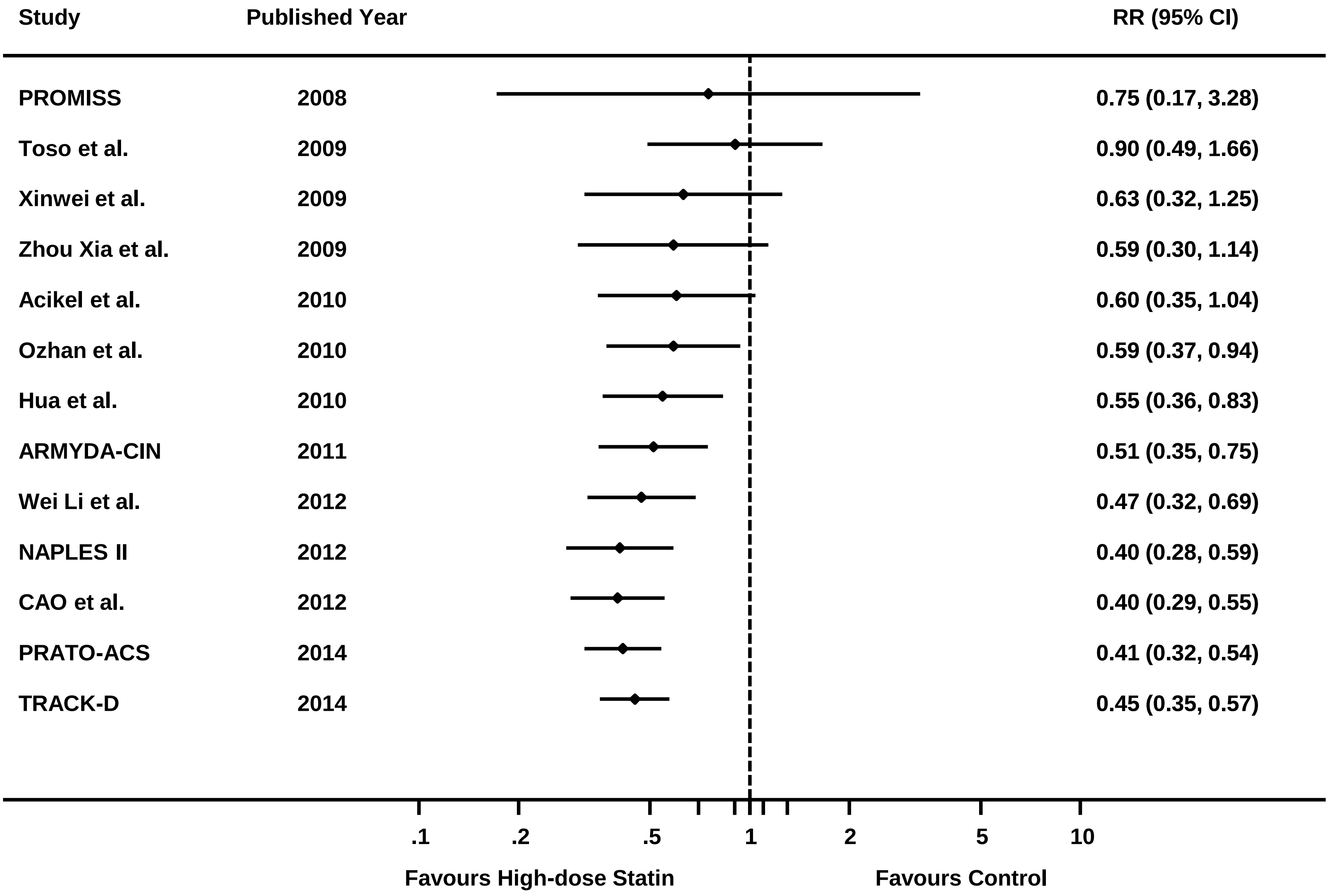

Figure S7. Random Effects Model of Change of Serum Creatinine

Study	Published Year	SMD (95% CI)	Statin N	Statin mean (SD), mg/dL	Control N	Control mean (SD), mg/dL	% Weight
PROMISS	2008	-0.08 (-0.33, 0.18)	118	.002 (0.16)	118	0.02 (0.23)	10.37
Toso et al.	2009	-0.07 (-0.30, 0.15)	152	0.07 (0.30)	152	0.09 (0.23)	10.70
Zhou Xia et al.	2009	-0.24 (-0.63, 0.15)	50	0.07 (0.24)	50	0.13 (0.26)	8.69
Acikel et al.	2010	-0.48 (-0.80, -0.17)	80	0.07 (0.15)	80	0.15 (0.18)	9.67
Ozhan et al.	2010	-0.39 (-0.74, -0.04)	60	-0.02 (0.13)	70	0.06 (0.25)	9.25
Hua et al.	2010	-0.29 (-0.59, 0.01)	76	0.27 (0.45)	97	0.41 (0.50)	9.82
ARMYDA-CIN	2011	-0.19 (-0.45, 0.06)	120	0.02 (0.35)	121	0.08 (0.27)	10.39
Wei Li et al.	2012	-0.92 (-1.24, -0.59)	78	0.13 (0.19)	83	0.34 (0.26)	9.54
CAO et al.	2012	-1.13 (-1.44, -0.81)	90	0.06 (0.19)	90	0.28 (0.20)	9.66
TRACK-D	2014	-0.03 (-0.10, 0.04)	1498	0.00 (0.29)	1500	0.01 (0.33)	11.91
Overall Random Effect Model		-0.37 (-0.59, -0.15)	2889		2936		100.0

Heterogeneity P < 0.001; I² = 88.3%
Test of Overall Effect Z = 3.29 (P = 0.001)

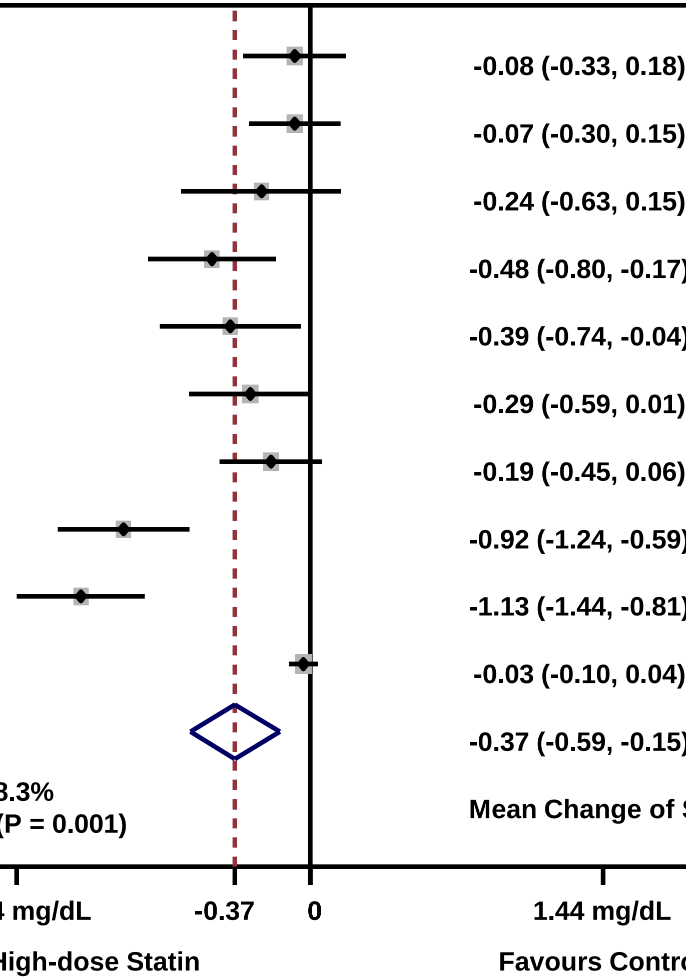

Mean Change of Serum Creatinine (mg/dL)

Figure S8. Subgroup According to Type of Contrast

Study	Published Year	RR (95% CI)	Events, Statin	Events, Control	% Weight
Iso-osmolar Contrast Agents					
PROMISS	2008	0.75 (0.17, 3.28)	3/118	4/118	2.66
Toso et al.	2009	0.94 (0.48, 1.83)	15/152	16/152	11.68
NAPLES II	2012	0.25 (0.12, 0.51)	9/202	37/208	10.69
PRATO-ACS	2014	0.45 (0.26, 0.77)	17/252	38/252	16.52
TRACK-D	2014	0.59 (0.39, 0.89)	34/1498	58/1500	25.02
Subtotal Effect (Z = 3.08, P = 0.002) Heterogeneity (P = 0.090 , I² = 50.2%)		0.52 (0.34, 0.79)	78/2222	153/2230	66.56
Low-osmolar Contrast Agent					
Xinwei et al.	2009	0.34 (0.14, 0.82)	6/113	18/115	7.00
Zhou Xia et al.	2009	0.14 (0.01, 2.70)	0/50	3/50	0.68
Acikel et al.	2010	0.33 (0.01, 8.06)	0/80	1/80	0.58
Ozhan et al.	2010	0.33 (0.07, 1.54)	2/60	7/70	2.47
Hua et al.	2010	0.40 (0.15, 1.04)	5/76	16/97	6.06
ARMYDA-CIN	2011	0.38 (0.15, 0.93)	6/120	16/121	6.76
Wei Li et al.	2012	0.16 (0.04, 0.70)	2/78	13/83	2.73
Subtotal Effect (Z = 4.72, P < 0.001) Heterogeneity (P = 0.962 , I² = 0.0%)		0.33 (0.21, 0.52)	21/577	74/616	26.28
Overall Random Effect Model		0.45 (0.35, 0.57)	105/2889	245/2936	100.00

Heterogeneity P = 0.364; I² = 8.2%
Test of Overall Effect Z = 6.44 (P < 0.001)

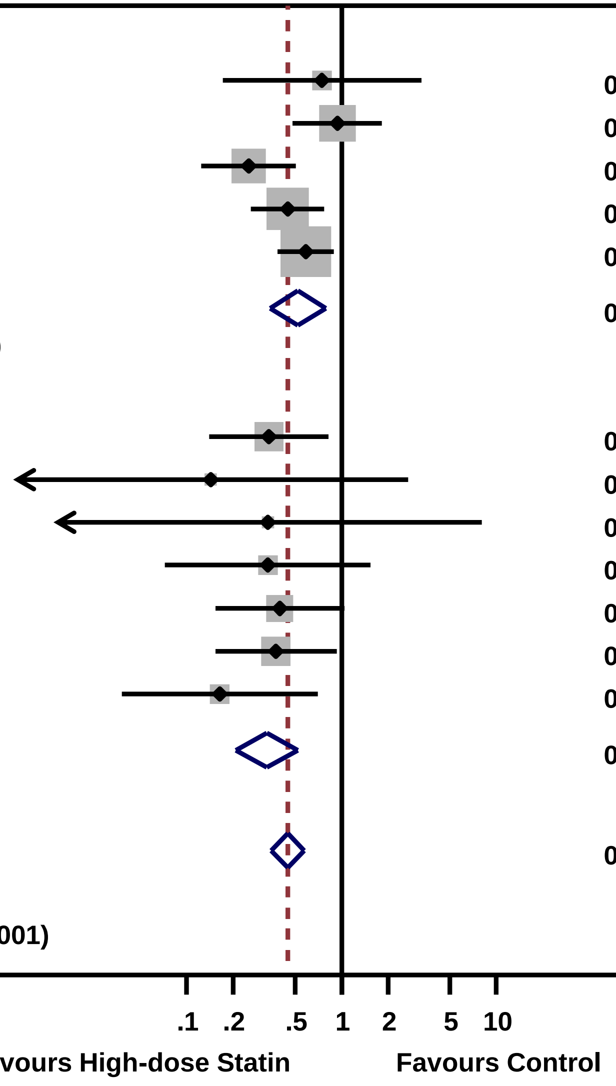

Figure S9. Subgroup According to Age of Patients

Study	Published Year	RR (95% CI)	Events, Statin	Events, Control	% Weight
Mean Age ≥ 60					
PROMISS	2008	0.75 (0.17, 3.28)	3/118	4/118	2.66
Toso et al.	2009	0.94 (0.48, 1.83)	15/152	16/152	11.68
Xinwei et al.	2009	0.34 (0.14, 0.82)	6/113	18/115	7.00
Zhou Xia et al.	2009	0.14 (0.01, 2.70)	0/50	3/50	0.68
Hua et al.	2010	0.40 (0.15, 1.04)	5/76	16/97	6.06
ARMYDA-CIN	2011	0.38 (0.15, 0.93)	6/120	16/121	6.76
Wei Li et al.	2012	0.16 (0.04, 0.70)	2/78	13/83	2.73
NAPLES II	2012	0.25 (0.12, 0.51)	9/202	37/208	10.69
CAO et al.	2012	0.33 (0.14, 0.80)	6/90	18/90	7.15
PRATO-ACS	2014	0.45 (0.26, 0.77)	17/252	38/252	16.52
TRACK-D	2014	0.59 (0.39, 0.89)	34/1498	58/1500	25.02
Subtotal Effect (Z = 5.76, P < 0.001)<br>Heterogeneity (P = 0.231 , I^2 = 22.3%)		0.44 (0.34, 0.58)	103/2749	237/2786	96.95
Mean Age < 60					
Acikel et al.	2010	0.33 (0.01, 8.06)	0/80	1/80	0.58
Ozhan et al.	2010	0.33 (0.07, 1.54)	2/60	7/70	2.47
Subtotal Effect (Z = 1.56, P = 0.119)<br>Heterogeneity (P = 1.000 , I^2 = 0.0%)		0.33 (0.08, 1.33)	2/140	8/150	3.05
Overall Random Effect Model		0.45 (0.35, 0.57)	105/2889	245/2936	100.00

Heterogeneity P = 0.364; I^2 = 8.2%
Test of Overall Effect Z = 6.44 (P < 0.001)

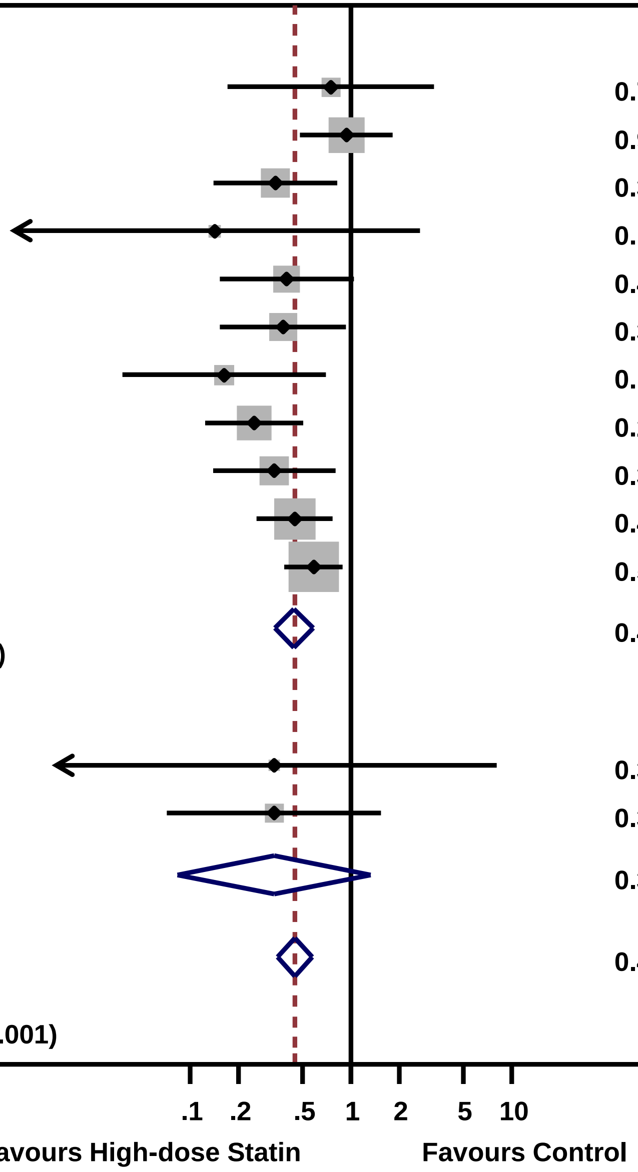

Figure S10. Subgroup According to Chronic Kidney Disease

Study	Published Year	RR (95% CI)	Events, Statin	Events, Control	% Weight
Studies with CKD patients (eGFR < 60 ml/min)					
PROMISS	2008	0.75 (0.17, 3.28)	3/118	4/118	2.66
Toso et al.	2009	0.94 (0.48, 1.83)	15/152	16/152	11.68
NAPLES II	2012	0.25 (0.12, 0.51)	9/202	37/208	10.69
TRACK-D	2014	0.59 (0.39, 0.89)	34/1498	58/1500	25.02
Subtotal Effect (Z = 2.10, P = 0.036)<br>Heterogeneity (P = 0.055 , I² = 60.5%)		0.55 (0.31, 0.96)	61/1970	115/1978	50.05
Without underlying CKD (eGFR ≥ 60 ml/min)					
Xinwei et al.	2009	0.34 (0.14, 0.82)	6/113	18/115	7.00
Zhou Xia et al.	2009	0.14 (0.01, 2.70)	0/50	3/50	0.68
Acikel et al.	2010	0.33 (0.01, 8.06)	0/80	1/80	0.58
Ozhan et al.	2010	0.33 (0.07, 1.54)	2/60	7/70	2.47
Hua et al.	2010	0.40 (0.15, 1.04)	5/76	16/97	6.06
ARMYDA-CIN	2011	0.38 (0.15, 0.93)	6/120	16/121	6.76
Wei Li et al.	2012	0.16 (0.04, 0.70)	2/78	13/83	2.73
CAO et al.	2012	0.33 (0.14, 0.80)	6/90	18/90	7.15
PRATO-ACS	2014	0.45 (0.26, 0.77)	17/252	38/252	16.52
Subtotal Effect (Z = 6.00, P < 0.001)<br>Heterogeneity (P = 0.973 , I² = 0.0%)		0.37 (0.27, 0.51)	44/919	130/958	49.95
Overall Random Effect Model<br>Heterogeneity P = 0.364; I² = 8.2%<br>Test of Overall Effect Z = 6.44 (P < 0.001)		0.45 (0.35, 0.57)	105/2889	245/2936	100.00

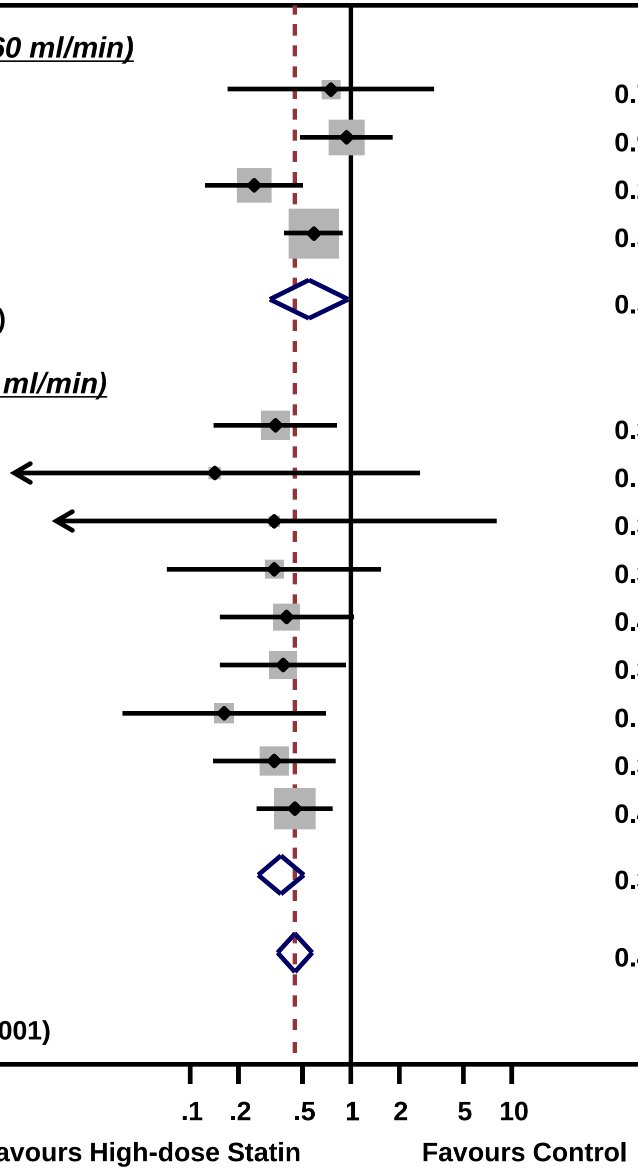

Figure S11. Subgroup According to Acute Coronary Syndrome

Study	Published Year	RR (95% CI)	Events, Statin	Events, Control	% Weight
Studies with Acute coronary Syndrome					
Xinwei et al.	2009	0.34 (0.14, 0.82)	6/113	18/115	7.00
ARMYDA-CIN	2011	0.38 (0.15, 0.93)	6/120	16/121	6.76
Wei Li et al.	2012	0.16 (0.04, 0.70)	2/78	13/83	2.73
CAO et al.	2012	0.33 (0.14, 0.80)	6/90	18/90	7.15
PRATO-ACS	2014	0.45 (0.26, 0.77)	17/252	38/252	16.52
Subtotal Effect (Z = 5.36, P < 0.001)<br>Heterogeneity (P = 0.776 , I² = 0.0%)		0.37 (0.26, 0.53)	37/653	103/661	40.16
Without Acute Coronary Syndrome					
PROMISS	2008	0.75 (0.17, 3.28)	3/118	4/118	2.66
Toso et al.	2009	0.94 (0.48, 1.83)	15/152	16/152	11.68
Zhou Xia et al.	2009	0.14 (0.01, 2.70)	0/50	3/50	0.68
Acikel et al.	2010	0.33 (0.01, 8.06)	0/80	1/80	0.58
Ozhan et al.	2010	0.33 (0.07, 1.54)	2/60	7/70	2.47
Hua et al.	2010	0.40 (0.15, 1.04)	5/76	16/97	6.06
NAPLES II	2012	0.25 (0.12, 0.51)	9/202	37/208	10.69
TRACK-D	2014	0.59 (0.39, 0.89)	34/1498	58/1500	25.02
Subtotal Effect (Z = 3.58, P < 0.001)<br>Heterogeneity (P = 0.236 , I² = 24.2%)		0.50 (0.34, 0.73)	68/2236	142/2275	59.84
Overall Random Effect Model<br>Heterogeneity P = 0.364; I² = 8.2%<br>Test of Overall Effect Z = 6.44 (P < 0.001)		0.45 (0.35, 0.57)	105/2889	245/2936	100.00

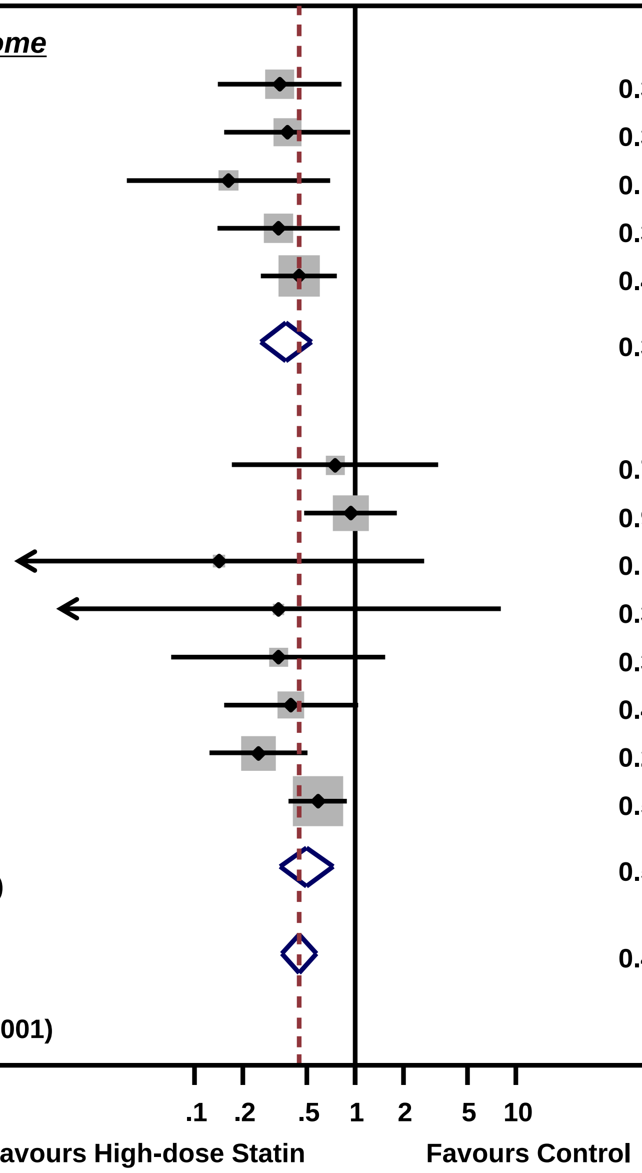

Figure S12. Subgroup According to N-acetylcystein

Study	Published Year	RR (95% CI)	Events, Statin	Events, Control	% Weight
With N-acetylcystein as adjunctive therapy					
Toso et al.	2009	0.94 (0.48, 1.83)	15/152	16/152	11.68
Ozhan et al.	2010	0.33 (0.07, 1.54)	2/60	7/70	2.47
NAPLES II	2012	0.25 (0.12, 0.51)	9/202	37/208	10.69
PRATO-ACS	2014	0.45 (0.26, 0.77)	17/252	38/252	16.52
Subtotal Effect (Z = 2.57, P = 0.010)<br>Heterogeneity (P = 0.058 , I² = 59.9%)		0.46 (0.25, 0.83)	43/666	98/682	41.35
Without N-acetylcystein as adjunctive therapy					
PROMISS	2008	0.75 (0.17, 3.28)	3/118	4/118	2.66
Xinwei et al.	2009	0.34 (0.14, 0.82)	6/113	18/115	7.00
Zhou Xia et al.	2009	0.14 (0.01, 2.70)	0/50	3/50	0.68
Acikel et al.	2010	0.33 (0.01, 8.06)	0/80	1/80	0.58
Hua et al.	2010	0.40 (0.15, 1.04)	5/76	16/97	6.06
ARMYDA-CIN	2011	0.38 (0.15, 0.93)	6/120	16/121	6.76
Wei Li et al.	2012	0.16 (0.04, 0.70)	2/78	13/83	2.73
CAO et al.	2012	0.33 (0.14, 0.80)	6/90	18/90	7.15
TRACK-D	2014	0.59 (0.39, 0.89)	34/1498	58/1500	25.02
Subtotal Effect (Z = 5.36, P < 0.001)<br>Heterogeneity (P = 0.695 , I² = 0.0%)		0.45 (0.34, 0.60)	62/2223	147/2254	58.65
Overall (I-squared = 8.2%, p = 0.364)<br>Heterogeneity P = 0.364; I² = 8.2%<br>Test of Overall Effect Z = 6.44 (P < 0.001)		0.45 (0.35, 0.57)	105/2889	245/2936	100.00

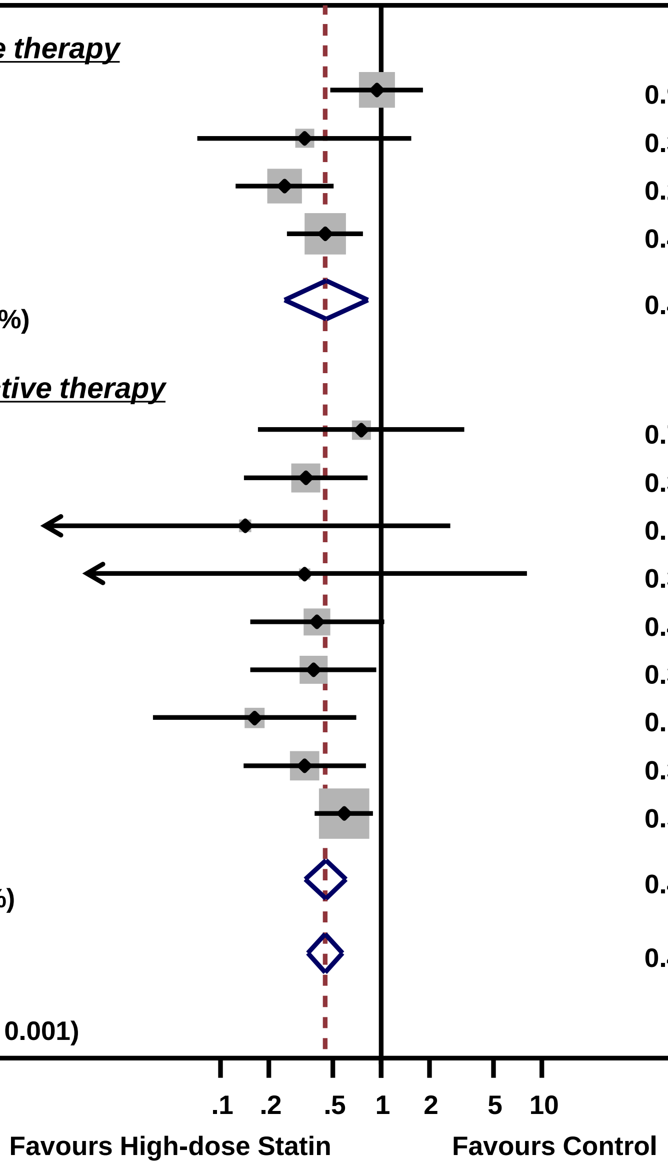

Supplement: File S1 — Supporting information files. Method S1, Search Strategy on Medline, EMBASE and Cochran Central. Method S2, Characteristics of the Excluded Study. Table S1, The Cochrane Collaboration's tool for assessing risk of bias. Figure S1, Risk of bias assessment graph. Risk of bias of each included trial was assessed with the Cochrane Collaboration's tool. This ‘risk of bias graph’ illustrates the proportion of studies with each of the judgments for each entry in the tool. Green represents ‘Yes (low risk of bias)’; yellow, ‘Unclear’; red, ‘No (high risk of bias)’. Figure S2, Risk of bias assessment summary. Risk of bias of each included trial was assessed with the Cochrane Collaboration's tool. This ‘risk of bias summary’ figure presents all of the judgments in a cross-tabulation of study by entry. Green represents ‘Yes (low risk of bias)’; yellow, ‘Unclear’; red, ‘No (high risk of bias)’. Figure S3, The effect of High-dose statin on the incidence of contrast-induced nephropathy by fixed effects model. Forest plot with relative risks for the incidence of contrast-induced nephropathy associated with high-dose statin versus low-dose statin or placebo for individual trials and the pooled population. The squares and the horizontal lines indicate the relative risks (by fixed effects model) and the 95% confidence intervals (CI) for each trial included; the size of each square is proportional to the statistical weight of a trial in the frequentist meta-analysis; diamond indicates the effect estimate derived from meta-analysis, with the center indicating the point estimate and the left and the right ends the 95% CI. Abbreviations: CI, confidence intervals; RR, relative risks. Figure S4, The effect of High-dose statin on mean change of post-procedural serum creatinine. Forest plot with standardized mean difference (SMD) for the mean change of post-procedural serum creatinine from the baseline value associated with high-dose statin versus low-dose statin or placebo for individual tria [file pone.0111397.s002.pdf]
